# New Permian radiolarians from east Asia and the quantitative reconstruction of their evolutionary and ecological significances

**DOI:** 10.1038/s41598-021-86262-7

**Published:** 2021-03-25

**Authors:** Yifan Xiao, Noritoshi Suzuki, Tsuyoshi Ito, Weihong He

**Affiliations:** 1 State Key Laboratory of Biogeology and Environmental Geology, China University of Geosciences, Wuhan, 430074 People’s Republic of China; 2 Department of Earth Science, Graduate School of Science, Tohoku University, Sendai, 980-8578 Japan; 3 Research Institute of Geology and Geoinformation, Geological Survey of Japan, AIST, Tsukuba, 305-8567 Japan

**Keywords:** Palaeontology, Evolutionary ecology

## Abstract

The biostratigraphically important Permian radiolarian genera *Pseudoalbaillella *sensu stricto and *Follicucullus* (Follicucullidae, Polycystinea) are discriminated by morphological gaps in their wings and segmentation. Previous statistical analyses demonstrated that *Longtanella* fills morphological gaps between these two genera. *Longtanella* has long been regarded as a junior synonym of *Parafollicucullus*, and only a few species have been described. Herein several true *Longtanella* species are recognized from South China, and eight new species and five indeterminate species are described and illustrated to prove the validity of the genus *Longtanella*. In addition, a new genus, *Parafollicucullinoides* gen. nov., is described. Their palaeogeographic distributions and living environments are explored by applying correspondence analysis (CA), with occurrence datasets of selected fusulinacean genera from the Japanese Islands, China and Sundaland. CA results indicate that *Longtanella* was present to a limited extent in warmer conditions in the fusulinacean Province B and C during Kungurian–Roadian time, and possibly lived above the thermocline and below the deepest limit of fusulinaceans. The *Pseudoalbaillella* and the *Follicucullus* group preferred open ocean conditions, living below the thermocline and distributed not only in the ‘Equatorial Warm Water Province’, but also the northern peri-Gondwana Cool Water Province and the southern North Cool Water Province.

## Introduction

The radiolarian order Albaillellaria is of particular importance because it includes index fossils for the Upper Devonian to the end-Permian^1^. Albaillellarians are characterized by poreless coverage with an internal bony triangular frame made of three intersecting rods and they are precisely identifiable at the species level thanks to rapid evolution. Their utility in biostratigraphy means that almost all albaillellarian morphotypes from the late Palaeozoic worldwide have been illustrated and described. Because members of the Permian Albaillellaria are also reported worldwide (South China^2^; Japan^3^; Thailand^4^; North American midcontinent^5^), biostratigraphic correlations in the uppermost Carboniferous and Permian can be conducted with high resolution across South China, Japan and Thailand, that roughly correspond to the fusulinacean Provinces B (Eastern Tethyan Province) and C (Panthalassan Province)^6^ established by Kobayashi^7^.

A well-studied albaillellarian group is the Cisuralian to Guadalupian family Follicucullidae Ormiston and Babcock, 1979^8^, which consists of more than 70 species. The Paleozoic Genera Working Group (PGW Group hereafter) decided to divide three genera, namely *Parafollicucullus*, *Follicucullus* and *Ishigaconus*, into 70 species^9,10^. Soon after this consensus was published, this three-genera scheme was undermined by data from the North America midcontinent^5^, China^11^ and Japan^3^. Xiao et al.^12^ re-evaluated the generic taxonomy of Follicucullidae and discussed their phylogenetic relationships using mathematical methods, such as Hayashi’s quantification theory II and parsimony theories. This study favoured these recent criticisms over the views of the PGW Group, and concluded on a ten-genera scheme, including the genus *Longtanella*^12^. Further, it not only confirmed the validity of *Longtanella* but also suggested that *Longtanella* is an important sister group in the evolution between the genera *Pseudoalbaillella *sensu stricto and *Follicucullus*. Just before this paper^12^ was accepted, the validity of *Longtanella* was newly morphologically confirmed^11^.

Herein, we investigate the validity of *Longtanella*, a key genus for understanding the evolution of late Palaeozoic radiolarians, but also for the validity of stratigraphic and palaeogeographic analyses through the Devonian to Permian. In order to fulfil these objectives, we reviewed all papers with illustrations of representatives of the Follicucullidae and examined their identifications with care. In exploring the recent suggestion^12^ that *Longtanella* could be a phylogenetic bridging genus between *Pseudoalbaillella *sensu stricto and the *Follicucullus* group (including all the species of *Follicucullus* and *Cariver*), we reexamined all the *Longtanella* morphotypes from South China. Then, we explored the palaeobioprovincial scheme of these three radiolarian genera together with fusulinacean genera by using correspondence analysis (CA). Comparisons of palaeogeographic distributions among *Longtanella* and its ancestor and descendent genera could be helpful in understanding the palaeobioprovincialism in radiolarians.

## Materials

### Geological and depositional setting

**Figure 1:**
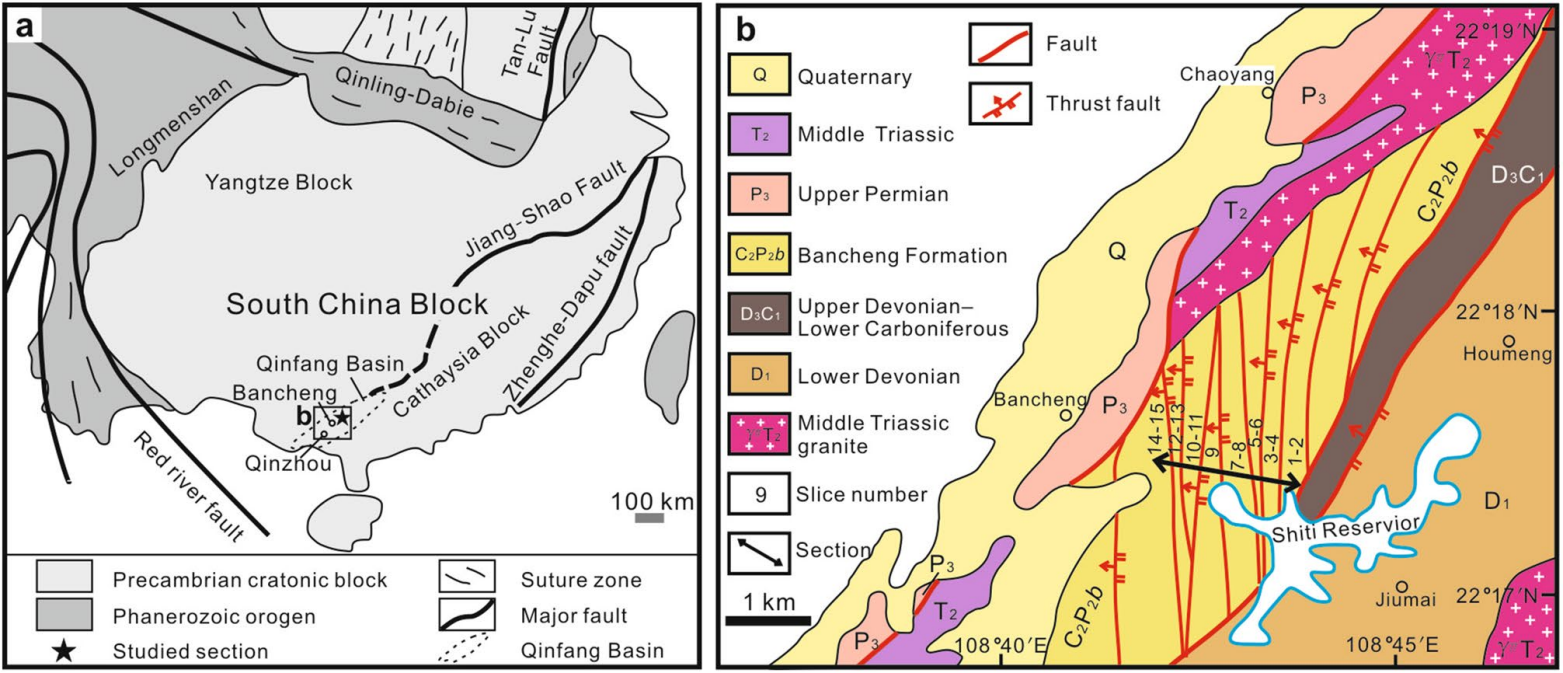
(**a**) Tectonic map of South China (revised after Zheng et al^13^) and location of the studied section. (**b**) Geological map of the studied area (revised after Ke et al ^14^). Y.F.X. created this figure using CorelDRAW X4 (https://www.coreldraw.com/en/pages/coreldraw-x4/).

We examined Cisuralian and Guadalupian bedded cherts of the Bancheng Formation in the Shiti section, Guangxi Province, China, and found 86 well- and moderately preserved specimens of 15 morphotypes whose morphological characters fit the definition of *Longtanella*. This section is located in the northern part of the Shiti Reservoir, about 4 km southeast of Bancheng Town, which is in the northern part of Qinbei District, Qinzhou City, Guangxi Province (Fig. 1a,b). The Bancheng Formation is dominated by thin-bedded chert, siliceous mudstone, carbonaceous mudstone and lithic greywacke. The Shiti section is divided into 15 tectonic slices by a series of thrust faults from east to west (Fig. 1b, supplement 1 Fig. S1). The total apparent thickness of the section is ca. 230 m, and the thickness of each slice ranges from 1 to 50 m except for poorly exposed parts. Folds with a short half wavelength are visible in some slices, but the continuity of each bed is traceable within a slice.

The Bancheng Formation was named by Zhong Keng and others in 1992 for the lithological body of greyish-yellow and greyish-red thin-bedded chert, radiolarian chert, muddy chert and mudstone near Bancheng Town^15^. The formation conformably overlies the Lower Carboniferous Shijia Formation and is unconformably overlain by Upper Permian or Lower Triassic strata^15–17^. The age of the Bancheng Formation ranges from Late Carboniferous to early Lopingian, and it yields abundant radiolarians, siliceous sponge spicules, a few conodonts, and foraminifera^17–19^. Most of the slices examined in this paper could be correlated to the Kungurian or Roadian on the basis of radiolarian index species (supplement 2), suggesting that the same horizon intervals are repeated along the section by thrust faults.

The depositional setting of these siliceous rocks is not a true ‘pelagic open-ocean’, but possibly a restricted ocean basin^20^ because true pelagic open-ocean chert as found in Japan have never yielded foraminifera. These rocks were deposited in the wedge-shaped Qinfang Basin^21^ which formed with the collision of the Yangtze block and the Cathaysia Block^20,22^ (Fig. 1a). The Qinfang Basin expanded as a deep-sea basinal setting in the Early Carboniferous and then it started to close as a bathyal sea during the Early to Middle Permian, and prior to the Late Permian the basin finally disappeared^23,24^.

### Palaeobioprovincial scheme

The most appropriate palaeobioprovincial scheme for these radiolarian genera (*Longtanella*, *Pseudoalbaillella *sensu stricto and the *Follicucullus* group) is the fusulinacean scheme. As radiolarians have never occurred with fusulinaceans in the same samples, this tendency is of interest.

Palaeobioprovincial analyses combining different taxa have rarely been performed for Permian marine organisms. It is generally understood that tropical bioprovinces were widely developed around the equator and expanded to higher latitudes in Greenhouse times such as the Eocene and Cretaceous^25^. The Permian, however, regardless of age and Greenhouse mode, experienced palaeobioprovinces that were partitioned by both latitude and longitude^26^; this phenomenon is shown by nektonic animals like conodonts, ammonoids and marine fishes^27–29^, and planktonic organisms are predicted to follow the similar patterns. Fossilized planktonic organisms in the Palaeozoic are limited to groups such as radiolarians whose skeleton is siliceous. We know that Cisuralian (Early Permian) radiolarian faunas were significantly different among the South Urals^30^, South China–Japan^2^, and North American midcontinent^31^. Lopingian (Late Permian) radiolarian faunas also showed strong provincialism between the Delaware Basin^32^ and the South China–Japan regions^33,34^. According to previous correlations, *Longtanella* ranges from UAZ_2_ (middle Asselian) to the end of UAZ_14_ (the Changhsingian) with relatively higher occurrence probability (*p* ≥ 0.20) in UAZ_6_ (Kungurian) and UAZ_7_ (Roadian)^6^.

**Figure 2:**
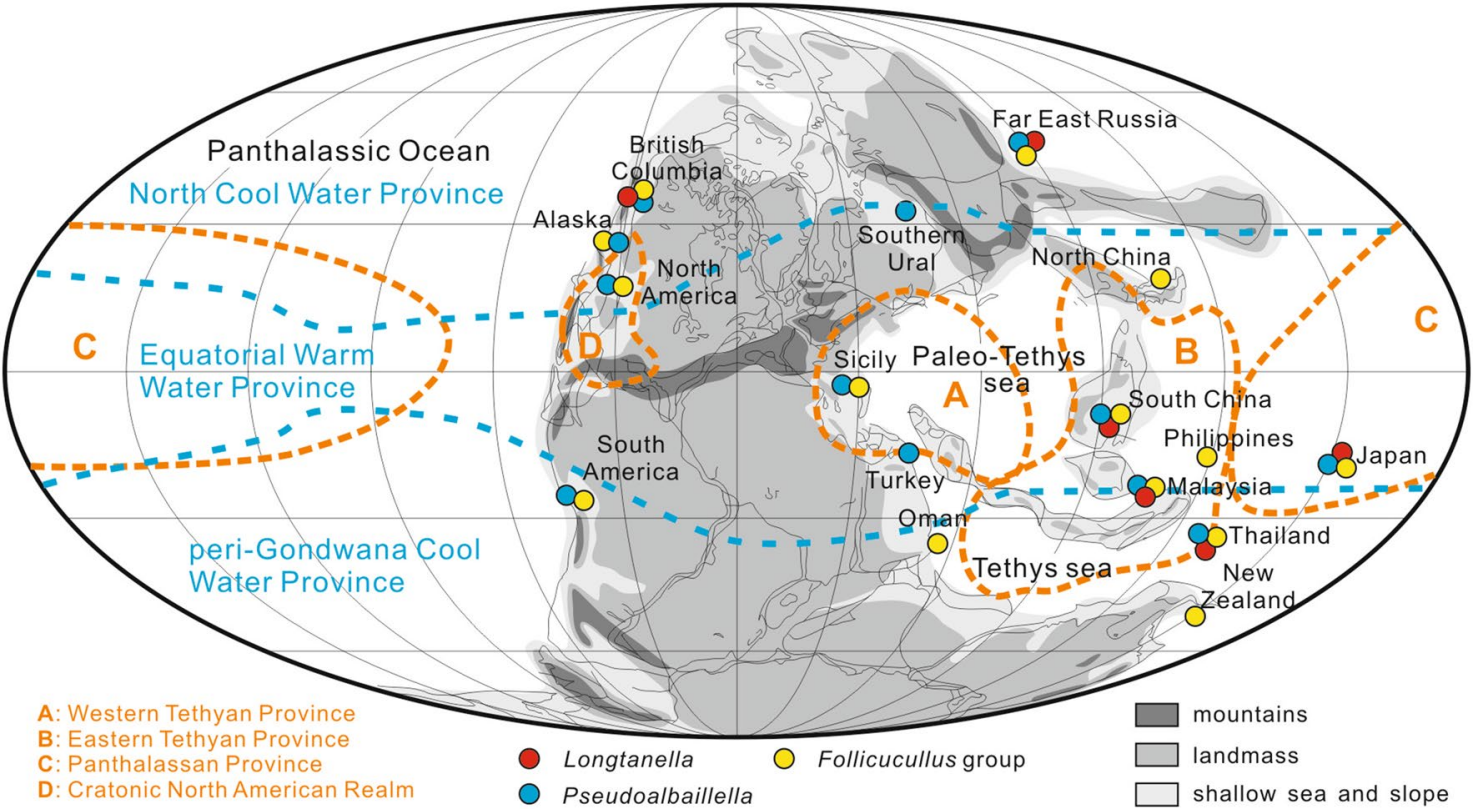
The global distributions of *Longtanella*, *Pseudoalbaillella* and the *Follicucullus* group. Palaeogeographical map revised after Lucas^35^. Orange lines show the fusulinid bioprovinces from Kobayashi^7^; blue lines show the conodont bioprovinces from Mei & Henderson^27^. Y.F.X. created this figure using CorelDRAW X4.

The metadatabase from our studies demonstrates that locations yielding *Longtanella* overlap with those of *Pseudoalbaillella* and the *Follicucullus* group in different ways in different palaeobioprovinces. The palaeogeographic distribution map shows that *Longtanella* are restricted to the fusulinacean Provinces B (Eastern Tethyan Province) and C (Panthalassan Province), whereas both *Pseudoalbaillella* and the *Follicucullus* group are reported not only in Provinces B and C, but also in Provinces A (Western Tethyan Province) and D (Cratonic North American Realm) (Fig. 2).

### Selected fusulinacean genera

The palaeogeography of fusulinaceans in east to southeast Asia including China and Japan has been well documented^36–38^. The Permian fusulinoidean palaeogeographic provinces in China are divided into the Cathaysia Tethys Province (CTP), Angara Tethys Province (ATP) and Gondwana Tethys Province (GTP)^39^. All these provinces constitute the Eastern Tethyan Province (Province B)^6,7^. In consideration of this knowledge, locality maps of fusulinaceans (occurrences of fusulinaceans are listed in supplement 3) were prepared for the selected seven genera (Table 1). These genera were chosen based on the facts that their taxonomic concepts are very stable, marked by obvious characters, and they are reliable guides to geographic distribution^26,40,41^.

**Table 1:**
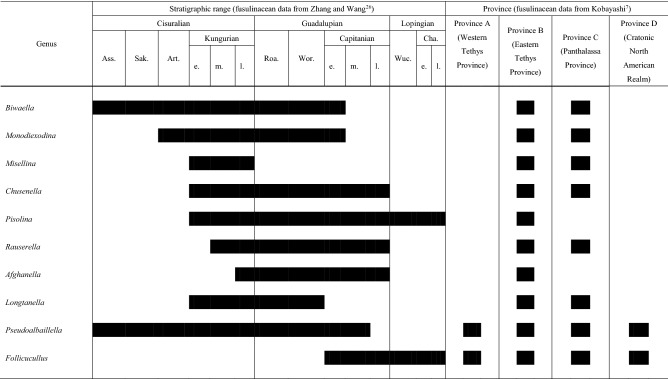
The stratigraphic range and province of the selected fusulinacean and radiolarian genera.

The faunal affinity between ‘cold-water’ *Monodiexodina* and ‘warm-water’ *Misellina* is often reported^37,38,42^, although these affinities seem to apply only for the Eastern Tethyan and Panthalassa provinces^43^ in the sense of Kobayashi^7^. *Chusenella* is helpful to indicate fusulinacean-bearing strata because this genus is commonly found in east and southeast Asia. Additional localities of Permian fusulinacean-bearing strata are provided by the distribution maps of *Biwaella* and *Rauserella*. Differing from the major fusulinacean province of Japan, the Panthalassan Province (Province C) and Eastern Tethyan Province (Province B) are marked by the presence of *Afghanella*, *Eopolydiexodina*, *Gallowaiina, Leella*, *Pisolina*, *Polydiexodina*, *Sumatrina*, *Wutuella* and *Zellia*^44,45^. Of these, *Afghanella* roughly overlaps the range of *Longtanella*.

## Results

### Occurrence of *Longtanella* and its age

#### Faunal composition and sample ages in Shiti section

**Figure 3:**
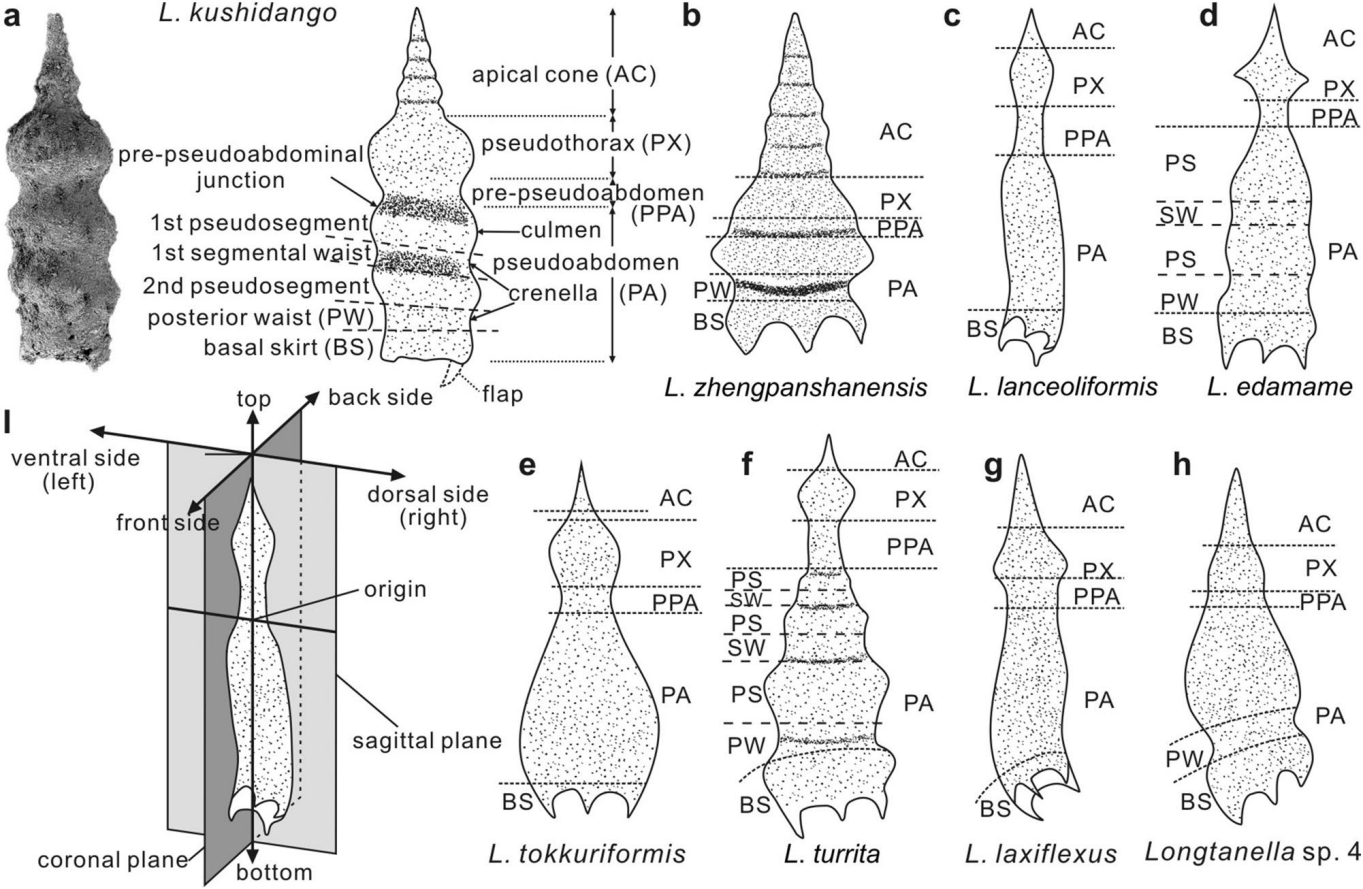
(**a–h**) Scanning electron microscopic image (**a**) and sketches showing the terminology of *Longtanella* species. (**i**) the diagram of the Cartesian coordinates. The morphological terminology was explained in supplement 4 and the descriptions of the species were in supplement 5. Y.F.X. created this figure using CorelDRAW X4. Photo credit: Y.F.X.

A total of 71 species belonging to 21 genera in the Shiti section have been identified from 38 of the 46 samples from the Bancheng Formation. This assemblage includes many common species for age determination such as *Albaillella asymmetrica* Ishiga & Imoto^46^, *Albaillella xiaodongensis* Wang^47^, *Parafollicucullus fusiformis* Holdsworth & Jones^48^, *Parafollicucullinoides globosus* (Ishiga & Imoto)^46^, *Parafollicucullinoides yanaharensis* (Nishimura & Ishiga)^49^. Besides, 15 morphotypes of *Longtanella* were recognized in the samples studied, including eight new species and five indeterminate species (Fig. 3, supplement 1 Figs S2–S5; supplement 5).

The age of the samples was determined by their coexistent species using the statistical likelihood UAZ ranges proposed recently ^6^. Most samples were correlated with UAZ_6_ or UAZ_7_ without contradiction in faunal association, except for samples ST9, ST8 and 13ST4-2. UAZ_6_ and UAZ_7_ are also correlated with the Kungurian and Roadian, respectively (supplement 2). All the *Longtanella* specimens are found in UAZ_6_ or UAZ_7_.

### Occurrences of related radiolarian genera

#### *Japanese Islands (supplement 1 *Fig. S6*)*

The basement rocks of the Japanese Islands comprise mainly Palaeozoic–Cenozoic accretionary complexes with island arc-related rocks^50,51^. Permian radiolarians occur in several Palaeozoic–Mesozoic geological units of the Japanese Islands: North Kitakami Belt, Mino–Tamba–Ashio Belt, Ultra-Tamba Belt, Maizuru Belt, Akiyoshi Belt, Hida-gaien Belt, North and South Chichibu belts and Kurosegawa Belt. *Pseudoalbaillella* and the *Follicucullus* group have been reported from everywhere in these tectonic belts except in the North Kitakami Belt, so the occurrences of *Longtanella* only are mapped herein. Occurrences of *Pseudoalbaillella* and *Longtanella* have been reported^52^ from the North Kitakami Belt of the Northeast Japan Zone. However, confirmed occurrences of the *Follicucullus* group have not been recognized yet, probably because of thermal metamorphism by Early Cretaceous granite^53^. There are no reliable Permian fusulinacean data, so we exclude the North Kitakami Belt from further discussion.

In the Inner Southwest Japan Zone, occurrences of *Longtanella* are restricted compared to *Pseudoalbaillella* and the *Follicucullus* group. *Pseudoalbaillella* and *Follicucullus* occur abundantly in the Mino–Tamba–Ashio Belt, but only a few occurrences of *Longtanella* have been reported^54^. *Longtanella* and *Pseudoalbaillella* were reported^55^ from the Hida-gaien Belt. No obvious *Longtanella* has been discovered from the Ultra-Tamba, Maizuru and Akiyoshi belts. However, *Longtanella* was noted^56^ in tuffaceous mudstone and chert of the Nagato Tectonic Zone. According to these authors, these rocks are possibly derived from the Akiyoshi Belt.

Relatively abundant *Longtanella* occur in the Outer Southwest Japan Zone. *Longtanella*, *Pseudoalbaillella* and the *Follicucullus* group co-occur in some sites of the Northern and Southern Chichibu belts^57,58^. The co-occurrence of *Longtanella*, *Pseudoalbaillella* and the *Follicucullus* group has been noted^59^ in the Kurosegawa Belt, which is located between the Northern and Southern Chichibu belts. Finally, *Longtanella* was discovered^60^ from Permian chert pebbles in the Lower Cretaceous Choshi Group of the Kurosegawa Belt.

#### Mainland China (exclusive of the Changning-Menglian Suture Zone) (supplement 1 Fig. S7)

The three radiolarian groups are quite common in the northern and western parts of South China, including Guangxi, Sichuan, Guizhou, Jiangsu, Hubei, and Anhui provinces, mainly from the Gufeng and Bancheng formations. Other occurrences are from accretionary complexes, such as the Xijinulan–Gangqiqu ophiolite complex and East Kunlun tectonic complex. *Longtanella* is found mostly from the ‘stable continental region’ in South China and less than *Pseudoalbaillella* and the *Follicucullus* group in numbers and distributions. The *Follicucullus* group is distributed widely, even in eastern Nei Mongol. Generally, these three groups are not so significantly different in distribution and are often associated with each other.

#### Sundaland and the Changning-Menglian Suture Zone (supplement 1 Fig. S8)

The tectonic continuity of the Changning-Menglian Suture Zone is closely related to tectonic zones in Sundaland (the continental core of SE Asia which is comprised of the Sunda shelf and parts of the Asian continental shelf), so the Changning-Menglian suture zone is considered here. *Longtanella* is not found in the Changning-Menglian Suture Zone where *Pseudoalbaillella* and the *Follicucullus* group exist^61,62^, but present in the Inthanon Suture Zone, the Sra Kaeo Suture and the Bentong-Raub Subzone. As discussed later, radiolarians are more limited in distribution than fusulinaceans. They almost all occur in suture zones except for *Pseudoalbaillella,* which is exclusively found from the Sukhothai Terrane and the Indochina Terrane. As with the distributions in South China, *Longtanella*, *Pseudoalbaillella* and the *Follicucullus* group often co-occurred, but there are more records of the latter two than *Longtanella*.

### Occurrences of selected fusulinacean genera

#### *Japanese Islands (supplement 1 *Fig. S6*)*

Occurrences of fusulinaceans in the Japanese Islands have been thoroughly plotted^63^. The fusulinacean zones that overlap those of the selected radiolarian genera are the *Pseudoschwagerina*, *Parafusulina* and *Neoschwagerina* zones in the sense of Toriyama^63^. The genus *Pisolina* was not reported in any of the Japanese Islands. *Monodiexodina* is exclusively found from the Hida-gaien and Kurosegawa belts. *Rauserella* is reported from the Akiyoshi, Mino-Tamba-Ashio and Northern and Southern Chichibu belts. *Afghanella* was only from the Akiyoshi Belt. *Biwaella* in the Japanese Islands is represented only by *Biwaella omiensis* Morikawa and Isomi, 1960^64^ from the Akiyoshi, the Mino-Tamba-Ashio, the Northern and Southern Chichibu belts. *Misellina* is the most widespread fusulinacean genus in Japan, having been illustrated from the Hida-gaien, Kurosegawa, Akiyoshi, Mino-Tamba-Ashio and the Northern and Southern Chichibu belts. Like *Misellina*, *Chusenella* has been found in the Akiyoshi, Kurosegawa, Mino-Tamba-Ashio, and Southern Chichibu belts, but not from the Hida-gaien Belt.

#### Mainland China (exclusive of the Changning-Menglian Suture Zone) (supplement 1 Fig. S7)

Carboniferous to Permian fusulinaceans are widely reported in China^45^ from ‘stable continental regions’ such as the Yangtze Craton and Cathaysia Fold Belt and ‘suture or accretionary complex zones’. *Pisolina* is mainly from ‘stable continental regions’, namely Anhui, Fujian, Guangxi, Guizhou, Hubei, Hunan, Jiangsu, Qinghai, Shaanxi, Sichuan, and Zhejiang provinces. Differing from the Japanese Islands, *Afghanella* was reported not only from ‘stable continental regions’, namely Guangxi, Guizhou, Hubei, Hunan, Sichuan provinces, but also from ‘suture or accretionary complex zones’ such as the Songpan-Ganzi Zone and the sutures between the North and South Qiangtang Blocks, namely Qinghai, Tibet, and Xinjiang provinces. A very few Permian *Biwaella* specimens are reported from Xinjiang, Guangxi, Guizhou, and Shaanxi provinces. *Rauserella* is widely known from several tectonic zones in Japan, but from limited areas in China, namely Anhui, Guizhou, Jiangsu, Jilin, and Sichuan provinces. *Monodiexodina* is illustrated from Tibet, Hainan, Heilongjiang, Hubei, Jiangsu, Jilin, Nei Mongol, and Sichuan provinces. Almost all fusulinacean localities are in the North China block where no radiolarian-bearing strata are recognized. *Misellina* is found everywhere in the Permian fusulinacean-bearing strata in China, but is not reported from Hainan, Jiangxi, Jilin, Heilongjiang, and Xinjiang provinces. All the provinces except for Hainan province are located in the North China block or adjacent tectonic zones. *Chusenella* is more diverse at the species level in China than in the Japanese Islands, and it is known from Tibet, Xinjiang, Anhui, Guangxi, Guizhou, Hubei, Hunan, Jiangsu, Jiangxi, Jilin, Qinghai, Shaanxi, and Sichuan provinces. It occurs at high species diversity in the South China block (Guangxi, Guizhou, Hubei, Hunan, Sichuan provinces) and Tibet.

#### Sundaland and the Changning-Menglian Suture Zone (supplement 1 Fig. S8)

The occurrence of fusulinaceans in Thailand and Malaysia was summarized by Toriyama^65^. *Pisolina* is known only from the Indochina Terrane, and *Biwaella* only from one locality of Thailand in the Sundaland and Changning-Menglian suture zone. *Rauserella* is also a minor component in Thailand and Yunnan. *Monodiexodina* is found from the Ailaoshan Suture and relevant sutures as well as the Inthanon Suture Zone. Highly diverse *Chusenella* was reported from Yunnan province, Myanmar and Thailand. *Afghanella* and *Misellina* are reported from limited localities in Yunnan. In contrast to rare *Afghanella* in Yunnan, diverse *Afghanella* species are reported from Thailand.

### Correspondence analysis (CA)

**Figure 4:**
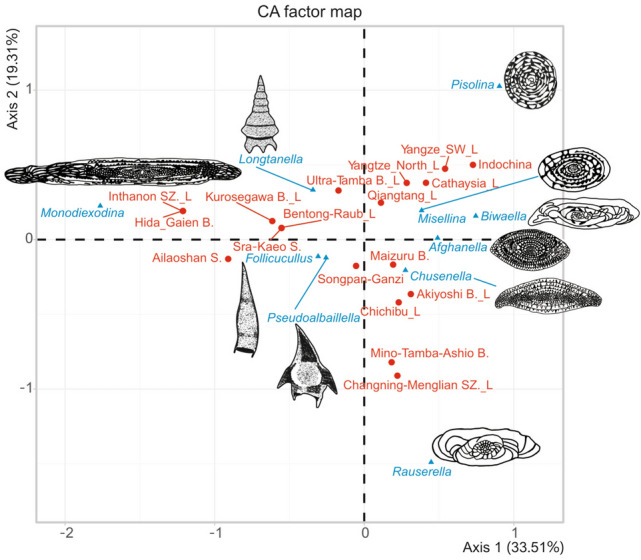
Correspondence plot of CA applied to the occurrences of selected radiolarian and fusulinid genera. Abbreviations: B., Belt; S., Suture; SZ., Suture Zone; L, indicates the presence of *Longtanella*. N.S. created this figure using R version 3.3.2 (https://www.r-project.org/) and Y.F.X. revised this figure using CorelDRAW X4.

The combinations of radiolarian and fusulinacean occurrences at the scale of tectonic divisions is so complex that we used CA to analyse the data. The analysis reduced the data to nine axes, of which the first three explain 69.2% of the variance (33.5%, 19.3% and 16.4% in ascending order; Table 2). On Axis 1, all the fusulinacean genera except *Monodiexodina* receive positive scores, whereas all the radiolarian genera receive negative scores (Fig. 4, Table 3). The highest absolute score on Axis 1 is negative, for *Monodiexodina* (− 1.77), and the highest positive scores are for *Pisolina* (0.90) and *Biwaella* (0.75). Values on Axis 2 are high (> 1.00) for *Pisolina* and low (< − 1.00) for *Rauserella* (Fig. 4). The inertia scores (Table 3) show that three genera (44.77% for *Monodiexodina*, 11.99% for *Biwaella* and 11.74% for *Pisolina*) explain 68.5% of the contributions to Axis 1. A total of 81.65% of Axis 2 is explained by *Rauserella* (55.32%) and *Pisolina* (26.23%). On Axis 3, high absolute scores are detected for *Afghanella* (− 0.741) and *Monodiexodina* (0.717) (Table 3), but the contributions to Axis 3 of these two genera is not high (28.21% for *Afghanella* and 15.09% for *Monodiexodina*), compared with other relatively higher contributions (19.21% for *Chusenella*, 16.99% for *Biwaella*, 14.74% for *Longtanella*) (Table 3). Based on Cos 2 (Table 3), two radiolarian genera (0.262 for *Pseudoalbaillella*, 0.368 for *Follicucullus*) and two fusulinacean genera (0.380 for *Biwaella* and 0.750 for *Monodiexodina*) are largely explained by Axis 1. Two fusulinacean genera (0.432 for *Pisolina* and 0.805 for *Rauserella*) are explained by Axis 2, and one radiolarian genus (0.269 for *Longtanella*) and two fusulinacean genera (0.386 for *Afghanella* and 0.532 for *Chusenella*) are explained by Axis 3.

**Table 2:** Eigen values and percentage variance of all axes.

Axis	Eigenvalue	Percentage of variance	Cummulative percentage of variance
Axis 1	0.309	33.5	33.5
Axis 2	0.178	19.3	52.8
Axis 3	0.151	16.4	69.2
Axis 4	0.105	11.4	80.6
Axis 5	0.076	8.3	88.9
Axis 6	0.039	4.3	93.1
Axis 7	0.029	3.1	96.2
Axis 8	0.026	2.8	99.0
Axis 9	0.009	1.0	100.0

**Table 3:**
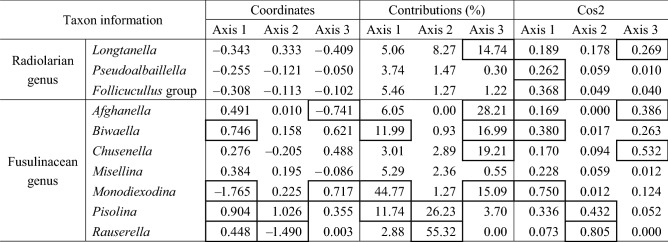
Coordinates, contributions and Cos2 of the columns (genus) in the first three dimensions.

Black box marks to bigger absolute values.

## Discussion

### *Longtanella* occurrence in references

Xiao et al.^6^ noted that *Longtanella* spp. was found between UAZ_2_ (middle Asselian) and UAZ_13_ (Wuchiapingian). Once Ito^11^ documented detailed morphological characters for *Longtanella*, the source data in Xiao et al.^6^ must be rechecked for *Longtanella*. Referring to Supplementary data 5 in Xiao et al.^6^, the most reliable range for *Longtanella* is revised as UAZ_6_ (Kungurian) to UAZ_8_ (Wordian). This difference was caused from our earlier incomplete knowledge of the ‘broken wing’. We conclude that the genus *Longtanella* is a good marker for the Kungurian to the Wordian.

### Interpretation of correspondence analysis

#### Axis 1

The positive direction of Axis 1 is largely contributed by *Pisolina* whereas the negative direction is contributed by *Monodiexodina* (Fig. 4, Table 3). The preferred palaeoenvironment of *Pisolina* is ‘semi-restricted platform facies’ which represents a low hydrodynamic environment^66^ or ‘inner gentle slope benthic biofacies’^67^. The preferred palaeoenvironment of *Monodiexodina* has been subject to two interpretations. One is an anti-tropical distribution^37^ although the suggested preferred temperatures are different from cold water^68^, middle latitude between high latitudinal cool/cold-water and tropical warm-water realms^37^, and temperate cool-water zone between temperate- and warm-water zones^43^ to warm water^69^. The other interpretation is high energy water conditions like clastic lithofacies^44^ and wave- and storm-reworked, transgressive lag deposits^70^. Considering the inner gentle slope benthic biofacies of *Pisolina*, the most likely interpretation of Axis 1 is a range from gentle water conditions (positive) to high-energy water conditions (negative). All three radiolarian genera are plotted in the negative area of Axis 1 (Table 3), probably reflecting open ocean conditions. *Pseudoalbaillella* and the *Follicucullus*-group are significant in Cos2 score (Table 3) although their contributions are not significant (Table 3). This also implies that open ocean conditions are not the most important factor for the distribution of the *Longtanella*.

#### Axis 2

The preferred palaeoenvironments of *Rauserella* have not been discussed; it occurs with abundant small foraminifers in intercoralite sediments^71^, abundant bryozoans with rare corals^72^, abundant non-colonial corals^73^, common calcareous algae^74^ and variable fragments of fossils^75^. Following this information, Axis 2 is defined by *Pisolina*-bearing limestone facies in the positive direction and *Rauserella*-bearing limestone facies in the negative direction. The selected three radiolarian genera have never been found from such limestones, and thus coordinates, contributions and Cos2 (Table 3) all are small scores, and not related to Axis 2.

#### Axes 3 and 4

As discussed above, *Monodiexodina* may have had an anti-tropical distribution. Preferred palaeoenvironments for *Afghanella* are poorly described in previous papers, but *Afghanella* in Zagros, Iran, occurs in a warming event^43^. The most likely interpretation of Axis 3 is an anti-tropical distribution in the positive direction and warmer conditions in the negative direction. The distribution of *Longtanella* is mainly described by Axis 3 (14.74 in Contribution, 0.269 in Cos2, Table 3) but also Axis 4 (21.79 in Contribution and 0.277 in Cos2), but the interpretation of Axis 4 is difficult. The coordinate score of Axis 3 for *Longtanella* is -0.409 (Table 3), suggesting warmer conditions on the line of anti-tropical to warmer conditions axis. Low values for *Pseudoalbaillella* and *Follicucullus* show that these two radiolarian genera were not related to this anti-tropical to warmer conditions axis.

### Summary of interpretations for selected radiolarian genera

The distributions of *Pseudoalbaillella* and *Follicucullus* correspond to open ocean conditions, but such conditions were not important for *Longtanella*. Instead, *Longtanella* preferred warmer condition along the anti-tropical to warmer conditions axis, but this condition did not impact on the distributions of *Pseudoalbaillella* and the *Follicucullus* group. Therefore, *Longtanella* appears to have been well adapted to warmer conditions, differing from the widespread ancestral *Pseudoalbaillella* as well as its widespread descendant the *Follicucullus* group. Compared to *Pseudoalbaillella, Longtanella* shows atrophied pseudothoracic wings and reduced pseudothorax and increased test height, and it evolved into the *Follicucullus* group by complete reduction of undulated segmentation of pseudoabdomen.

### Revisiting the palaeogeographic map

The fusulinacean palaeogeographic map shows different palaeo-provinces among the western Palaeo-Tethys (Province A), eastern Meso-Tethys and Meso-Tethys (Province B), Panthalassa (Province C) and the eastern margin of Panthalassa (Province D)^7^ (Fig. 1). Our CA analysis shows the combination of Provinces B and C with a common distribution over this area for *Pseudoalbaillella* and the *Follicucullus* group. This result also supports the earlier recognition^6^ of the same low-latitudinal standard Permian radiolarian biostratigraphy between Provinces B and C. The relatively restricted distribution of *Longtanella* in Provinces B and C is explained by the preferred warmer conditions in our CA. This seems to contradict the higher latitudinal distribution in British Columbia and Far East Russia. However, the original depositional position of parts of the British Columbia blocks or terranes were at a northern middle latitude 200 Ma age (earliest Jurassic)^76^ and the original position in the Permian was probably at a lower latitude. Permian radiolarian and fusulinacean localities in Far East Russia are a northern extension of the tectonic divisions of the Japanese Islands^50^ and thus the original depositional positions were also at low latitudes. The Permian radiolarian faunas are the same between Japan and Far East Russia^6^. The fusulinacean faunal similarity among British Columbia, Far East Russia (= Primorye, Sikhote-Alin) and the Japanese Islands is already known^77^ and was identified as the ‘Tethyan-Panthalassa fauna’ or ‘*Yabeina* territory’ in subsequent studies^71,78,79^. In consideration of these tectonic and faunal affinities, *Longtanella* may be present in a limited way in warmer conditions in the fusulinacean Provinces B and C. For conodonts, the Permian ‘Equatorial Warm Water Province’ along the zone between northern and southern mid-latitudes^27^ (Fig. 1) includes all of fusulinacean Provinces A, B and C. Both *Pseudoalbaillella* and the *Follicucullus* group are distributed not only in the ‘Equatorial Warm Water Province’ but also in the northern peri-Gondwana Cool Water Province and southern North Cool Water Province. By contrast, *Longtanella* is distributed in a very limited way, as explained above.

The terrestrial and oceanic realms were mapped in the latest Permian^80^. Because the Middle Permian was cooler than the latest Permian^81^, the tropical ocean zone was narrower in the Middle Permian^80^. Waters above the thermocline are variable whereas water below the thermocline is homogenously cold in low to middle latitudes^82,83^. This oceanic rule requires the warm-water-dependent *Longtanella* to live above the thermocline, which means that it cannot be associated with fusulinaceans because they lived in deep-water environments. In the maps of Middle Permian palaeoprovinces with brachiopods^84^, the distributions of both *Pseudoalbaillella* and the *Follicucullus* group cover not only the Cathayasian realm but also the southern part of the Sino-Mongolian realm and part of the Cimmerian realm, just like the conodont palaeoprovinces^27^. The distributions of brachiopods and conodonts are largely controlled by latitudinal surface water temperature (SST), but the differences in distribution of *Pseudoalbaillella* and the *Follicucullus* group suggest that their distribution did not depend on SST. These two genera are found in the Middle Permian limestone in the Guadalupe and Apache mountains of West Texas. Noble et al. ^85^ explained that a stimulatory response to increased runoff is related to abundance changes in *Follicucullus ventricosus*. Such runoff conditions generate a similar oceanographic situation to an upwelling region in terms of oceanic physiology^86^ and deep-water radiolarians can then be found in the intermediate zone^87^. Xiao et al.^88^ defined water depths scheme for the Permian referred from the modern oceanography. The thermocline is roughly situated in similar water depth to the deep chlorophyll maximum (DCM)” and we defined the very shallow and shallow zones by the DCM. As *Longtanella* lived in different water conditions from *Pseudoalbaillella* and the *Follicucullus* group, the latter would have lived below the thermocline.

### Evolutionary perspectives

**Figure 5:**
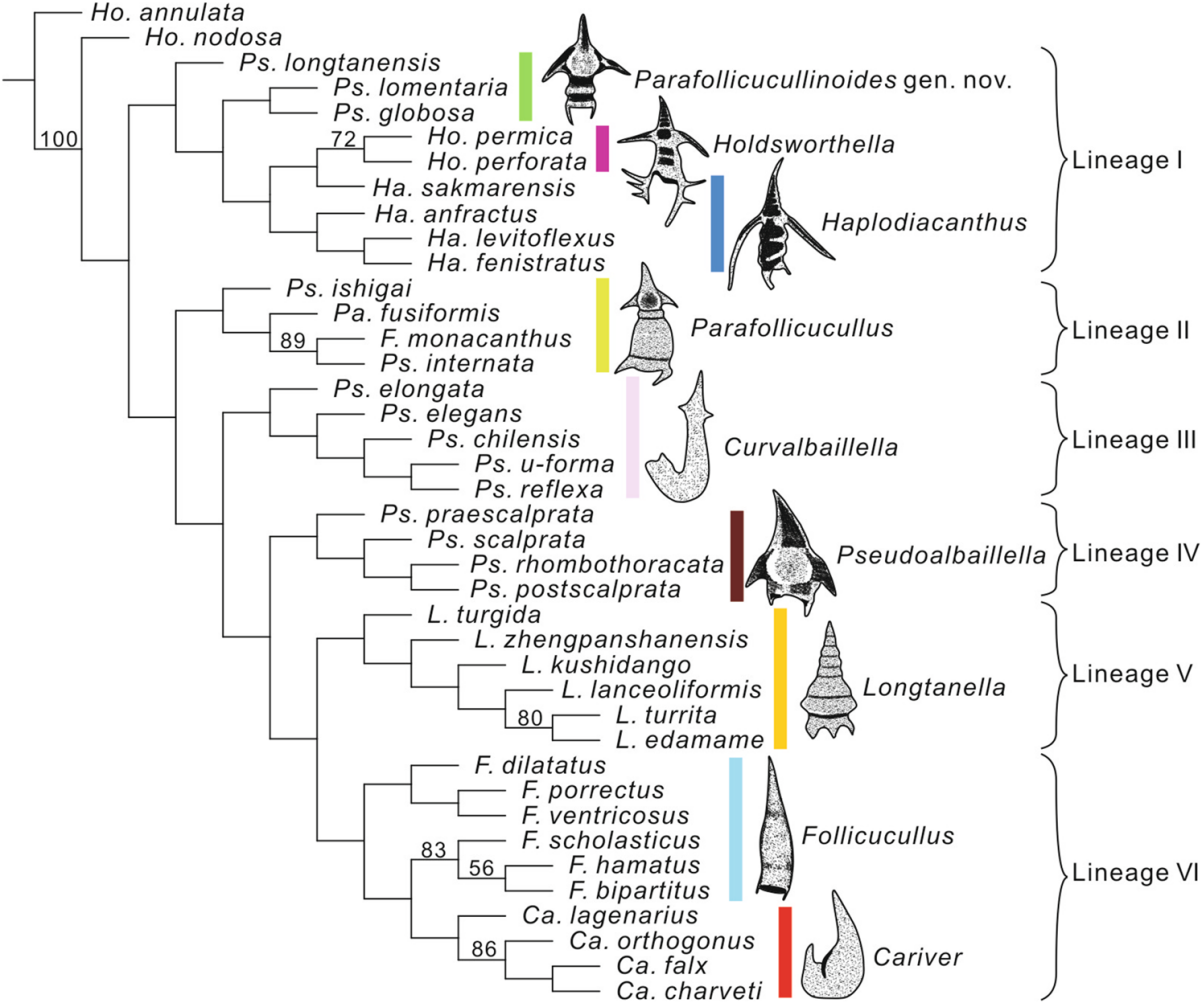
Phylogenetic tree of Follicucullidae obtained by the parsimony analysis (revised after Xiao et al.^11^, the character data set is provided in supplement 6). The species name is written as the original described name. Genus abbreviations: *L.*, *Longtanella*; *Pa.*, *Parafollicucullus*; *Ha.*, *Haplodiacanthus*; *Ho.*, *Holdsworthella*; *Ps.*, *Pseudoalbaillella*; *F.*, *Follicucullus*; *Ca.*, *Cariver*. Y.F.X. created this figure using TNT version 1.5 (http://www.lillo.org.ar/phylogeny/tnt/) and CorelDRAW X4.

Xiao et al.^6^ showed mathematically that *Longtanella* evolved from *Pseudoalbaillella*, whereas the *Follicucullus* group was divergent from *Longtanella* (Fig. 5). Our study herein first recognized that *Longtanella* was limited to Provinces B and C in the Kungurian to Wordian, differing from provinces where its ancestor (*Pseudoalbaillella*) and descendant (*Follicucullus*) lived. The short range and limited distribution of *Longtanella* might be related to some climatic changes. Although our data are insufficient to specify this prediction, the early Kungurian is known for the end of late Artinskian–early Kungurian warming and maximum marine flooding event in east Gondwana^89^ and the early Capitanian is the time of diversification of *Follicucullus* species^90^. We hypothesized that *Longtanella* evolved near east Gondwana to adapt to warmer waters with less input of fresh water around the early Kungurian and lost the competition for survival against the newly diversified *Follicucullus* species around the early-middle Capitanian.

## Conclusions

Eight new species and five indeterminate species of *Longtanella* are described, which greatly expands knowledge of this genus. Correspondence analysis was applied to occurrences of *Longtanella*, its sister taxa (*Pseudoalbaillella* and the *Follicucullus* group), and seven fusulinacean genera. The most likely interpretation of the CA output Axis 1 is gentle (positive) to high-energy water conditions (negative). Axis 2 is defined by *Rauserella*-bearing (negative) to *Pisolina*-bearing limestone facies (positive). Axis 3 is warmer conditions (negative) to anti-tropical distribution (positive).

Comparisons with fusulinacean palaeoenvironmental interpretations based on CA suggest that there are some differences in the distributions of *Longtanella*, *Pseudoalbaillella* and the *Follicucullus* group. *Pseudoalbaillella* and the *Follicucullus* group favour open ocean conditions, but this condition is not important for *Longtanella*. *Longtanella* may be present in a limited way in warmer conditions in the fusulinacean Provinces B and C. *Pseudoalbaillella* and the *Follicucullus* group are distributed in not only the ‘Equatorial Warm Water Province’ but also in the northern peri-Gondwana Cool Water Province and southern North Cool Water Province in the conodont scheme. *Longtanella* lived above the thermocline and below the deepest limitation of fusulinaceans. *Pseudoalbaillella* and the *Follicucullus* group lived below the thermocline.

This leads to the suggestion that *Longtanella* was well adapted to warmer conditions, differing from the widespread ancestral *Pseudoalbaillella* and the widespread descendant *Follicucullus*. The evolutionary appearance of *Longtanella* contributed to the atrophy of pseudothoraxic wings and size of the pseudothorax and lengthening of the total height of the test from *Pseudoalbaillella* and evolved to the *Follicucullus* group by complete reduction of undulated segmentation of the pseudoabdomen. The appearance of *Longtanella* may relate to a regional warmer event after the early Kungurian glacial period in East Gondwana and its extinction is likely related with diversification of the *Follicucullus* group in the early-middle Capitanian.

## Methods

### Sampling and analysis procedures

Forty-six samples were collected from all slices of the Bancheng Formation in the Shiti section. The fragmented samples were soaked in a 4% HF solution for 10 h at room temperature. After discarding the supernatant liquid, the acid residues were transferred to other containers, then water was added until the residues were greatly diluted. After this step, the same steps were repeated more than 40 times for each sample. Disaggregated particles were wet-sieved through a 54 µm mesh sieve and dried at the end of 40 time steps. By preliminary observation under a binocular microscope, 38 samples with rich identifiable radiolarians were selected. Individuals with distinguishable morphological characters were picked for species identification under a binocular microscope, and then better-preserved specimens (over 800 specimens in total) were photographed under the scanning electron microscope (Hitachi SU8010 in State Key Laboratory of Biogeology and Environmental Geology, China University of Geosciences) for further morphological examination and species identification.

### Meta-database

Detailed occurrences of selected fusulinacean and radiolarian genera were mapped in the Japanese Islands, China and Sundaland. Sundaland includes the southern to southwestern parts of China, Vietnam, Thailand, eastern Myanmar, Indonesia and Malaysia. Global distribution maps for the selected radiolarian genera include all other regions in the world. Following the current concepts of radiolarian genera, *Longtanella*, *Pseudoalbaillella *sensu stricto and the *Follicucullus*-group (*Follicucullus* and *Cariver*)^12^ were re-identified from illustrated specimens in publications that are archived in Tohoku University (ca. 5800 papers).

The world palaeogeographic map was drawn based on the Middle to Late Permian tectonic reconstruction map (Fig. 2). Both the *Pseudoalbaillella* and *Follicucullus* group are widely distributed in the western and eastern margins of Palaeo-Tethys (Sicily^91^; Turkey^92^; South China^2^; Malaysia^93^), east of the Meso-Tethys (Thailand^94^), western Panthalassan Ocean (Far East Russia^95^; Japan^58^; New Zealand^96^), eastern Panthalassan Ocean (British Columbia^97^; Alaska^48^; South America^98^). By contrast, known occurrences of *Longtanella* are limited to the western Panthalassan Ocean, eastern margin of Palaeo-Tethys, east of Meso-Tethys, and part of the eastern Panthalassan Ocean. This suggests a more limited distribution for *Longtanella* than its ancestral *Pseudoalbaillella* or descendent *Follicucullus* group. The middle to upper Permian marine deposits with radiolarians in the Japanese Islands, China and Sundaland have been well studied since the 1980s. In these areas, any identifiable morphotypes without a taxonomic name are regularly illustrated, and study of these indicates the occurrence of *Longtanella* at the level of tectonic divisions.

### Statistical analysis

Statistical analyses of radiolarian palaeobioprovinces were performed with the occurrence or absence dataset (binary data) of co-occurrences of radiolarian and fusulinacean genera at the level of tectonic belts. Questionable occurrences or questionable identifications were not included in the occurrence list. The tectonic divisional scheme comes from published work for the Japanese Islands^51^, China^13^, and Sundaland^99^.

For the present studies, we used CA (the dataset for CA is in supplement 7). CA is one of the most useful multivariate statistical methods to explore occurrence and absence data^88^. In consideration of taxonomic stability at the genus level, the occurrences of seven fusulinacean genera in the Early-Middle Permian from China, Japan and Sundaland were compiled to analyze possible factors in the distribution of *Longtanella*. The CA suggests that *Longtanella* differs from the *Pseudoalbaillella* and the *Follicucullus* group not only in palaeogeographic distribution, but also in its preferred temperature living conditions. The CA, in short, can output independent factors that describe distributions.

The CA was performed with the statistical software R (R ver. 4.0.2) and plugins (RStudio ver. 1.3, Rcmdr ver. 2.7–0, RcmdrPlugin.EZR ver. 1.52, RcmderPlugin.FactoMineR ver 1.7).

## Supplementary Information

Supplementary Information
