# Supplementary material for: New Permian radiolarians from east Asia and the quantitative reconstruction of their evolutionary and ecological significances
Source: Sci Rep. 2021 Mar 25;11:6831. doi: 10.1038/s41598-021-86262-7 (PMC7994911; doi:10.1038/s41598-021-86262-7)

**Supplement material-**

**New Permian radiolarians from east Asia and the quantitative reconstruction of their evolutionary and ecological significances**

**Yifan Xiao**^1^*, **Noritoshi Suzuki**^2^, **Tsuyoshi Ito**^3^, **Weihong He**^1^

*^1^State Key Laboratory of Biogeology and Environmental Geology, China University of*

*Geosciences, Wuhan 430074, PR China;*

*^2^Department of Earth Science, Graduate School of Science, Tohoku University, Sendai 980-8578, Japan;*

*^3^Research Institute of Geology and Geoinformation, Geological Survey of Japan, AIST, Tsukuba 305-8567, Japan.*

*Corresponding author. Email: [yifanxiao@cug.edu.cn](mailto:yifanxiao@cug.edu.cn)

**Content**

**Supplement 1. Supplement figures.**

Fig. S1 Cross-section of the Bancheng Formation, Shiti section, Guangxi province, China.

Figs S2–S5 *Longtanella* species from the Shiti section, Bancheng Formation.

Fig. S6 Occurrences of the selected fusulinacean genera and Longtanella in Japanese Islands (Tectonic map revised after Wallis et al. 2020).

Fig. S7 Occurrences of the selected fusulinacean and radiolarian genera in Mainland China (Tectonic map revised after Zheng et al. 2013).

Fig. S8 Occurrences of the selected fusulinacean and radiolarian genera in Sundaland (Tectonic map revised after Metcalfe 2013).

**Supplement 2.** **UAZ range (age) of each sample in the Shiti section decided by radiolarian species.**

**Supplement 3. Occurrences of fusulinaceans and radiolarians.**

**Supplement 4.** **The morphological terminology for the albaillellids in Figure 3, Supplement 5 and 6.**

**Supplement 5. Systematic Palaeontology.**

**Supplement 6. The character data set used for phylogenetic tree of Follicucullidae.**

**Supplement 7. Meta dataset for correspondence analysis (CA)**

**Supplement 1. Supplement figures.**


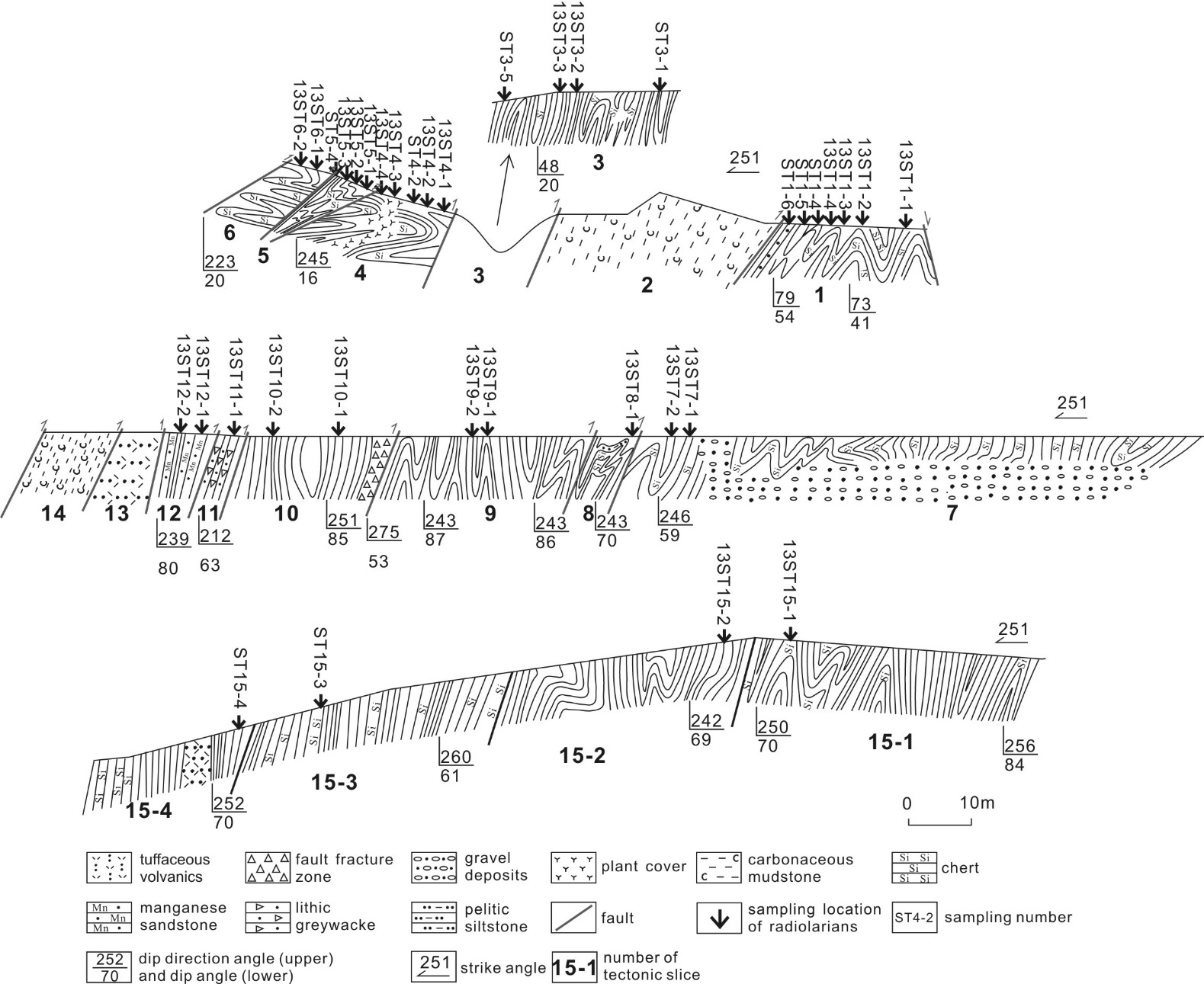


**Figure S1.** Cross-section of the Bancheng Formation, Shiti section, Guangxi province, China. W.H.H. created this figure using CorelDRAW X4.





**Figure S2.** *Longtanella* species from the Shiti section, Bancheng Formation. Scale bar equals 100 μm for all figures, except from N (50 μm). **A–P,** *Longtanella lanceoliformis* Xiao & Suzuki sp. nov. **A,** 13ST9-1_012; **B,** 13ST9-1_014; **C,** 13ST9-1_086; **D,** paratype, ST1-2_i001; **E,** ST1-2_i015; **F,** 13ST9-1_i003; **G,** ST1-2_i028; **H,** ST1-2_i030; **I,** ST1-2_i029; **J,** 13ST9-1_013; **K,** ST1-2_i031; **L,** 13ST9-1_087; **M,** holotype, ST 1-2_i026; **N,** ST 1-2_i026; **O,** ST1-2_i003; **P,** ST1-2_i032. **Q–X,** *Longtanella edamame* Xiao & Suzuki sp. nov. **Q,** holotype, 13ST15-4_148; **R,** 13ST6-1_010; **S,** 13ST15-4_149; **T,** ST1-3_i060; **U,** ST6-1_i001; **V,** 13ST6-1_248; **W,** paratype, 13ST6-1_245; **X,** 13ST6-1_250. **Y, Z,** *Longtanella* sp. 1. **Y,** ST6-1_i008; **Z,** ST9-1_i020. Photo credit: Y.F.X.





**Figure S3.** *Longtanella* species from the Shiti section, Bancheng Formation. Scale bar equals 100 μm for all figures. **A–C,** *Longtanella* sp. cf. *Longtanella edamame* Xiao & Suzuki sp. nov. **A,** ST1-2R_006; **B,** ST1-3_054; **C,** ST1-3_055. **D–G,** *Longtanella jingyi* Xiao & Suzuki sp. nov. **D,** paratype, ST1-3_061; **E,** ST1-3_i025; **F,** holotype, ST1-3_i053; **G,** ST1-3_i039. **H–O,** *Longtanella tokkuriformis* Xiao & Suzuki sp. nov. **H,** 13ST1-4_004; **I,** 13ST1-4_005; **J,** paratype, 13ST1-4_072; **K,** 13ST1-4_i005; **L,** holotype, ST1-3_i045; **M,** ST1-6_i011; **N,** ST1-3_i030; **O,** ST3-5R_036. **P–U,** *Longtanella turrita* Xiao & Suzuki sp. nov. **P,** ST1-3_049; **Q,** paratype, ST1-3_048; **R,** holotype, ST1-3_052; **S,** ST1-3_050; **T,** ST1-3_051; **U,** ST1-3_053. **V**–**W**: *Longtanella* sp. 2. **V,** 13ST15-4_153; **W,** ST1-3_i047. Photo credit: Y.F.X.





**Figure S4.** *Longtanella* species from the Shiti section, Bancheng Formation. Scale bar equals 100 μm for all figures. **A–N,** *Longtanella* *kushidango* Xiao & Suzuki sp. nov. **A,** ST9-1_i001; **B,** ST9-1_i002; **C,** ST9-1_i003; **D,** ST9-1_i004; **E,** holotype, ST9-1_i005; **F,** ST9-1_i012; **G,** ST9-1_i007; **H,** paratype, ST9-1_i006; **I,** ST9-1_i009; **J,** ST9-1_i013; **K,** ST9-1_i013; **L,** ST9R_178; **M,** ST9-1_i021; **N,** ST9R_181. **O, P,** *Longtanella*? sp. 3. **O,** ST1-2_i006; **P,** ST1-2_i035. **Q–U,** *Longtanella laxiflexus* Xiao & Suzuki sp. nov. **Q,** paratype, 13ST9-1_083; **R,** ST1-1_i001; **S,** ST1-1_i002; **T,** 13ST9-1_i004; **U,** holotype, ST1-2_i011; **V, W,** *Longtanella* sp. cf. *Longtanella laxiflexus* Xiao & Suzuki sp. nov. **V,** 13ST9-1_i007; **W,** 13ST9-1_i002. Photo credit: Y.F.X.





**Figure S5.** *Longtanella* species from the Shiti section, Bancheng Formation. Scale bar equals 100 μm for all figures. **A–G,** *Longtanella* *follicucullinoides* Xiao & Suzuki sp. nov. **A,** ST1-2_i025; **B,** ST1-2_i013; **C,** ST1-2_i005; **D,** ST1-2_i016; **E,** ST1-2_i010; **F,** holotype, ST1-2_i007; **G,** paratype, ST1-2_i022. **H–J,** *Longtanella* sp. 4. **H,** ST1-6_i006; **I,** ST1-6_i004; **J,** ST6-1_i005. **K–P,** *Longtanella* sp. 5. **K,** ST1-2_i008; **L,** ST1-3_i026; **M,** ST1-3_i032; **N,** ST1-3_i033; **O,** ST1-2_i004; **P**, 13ST6-1_257. Photo credit: Y.F.X.


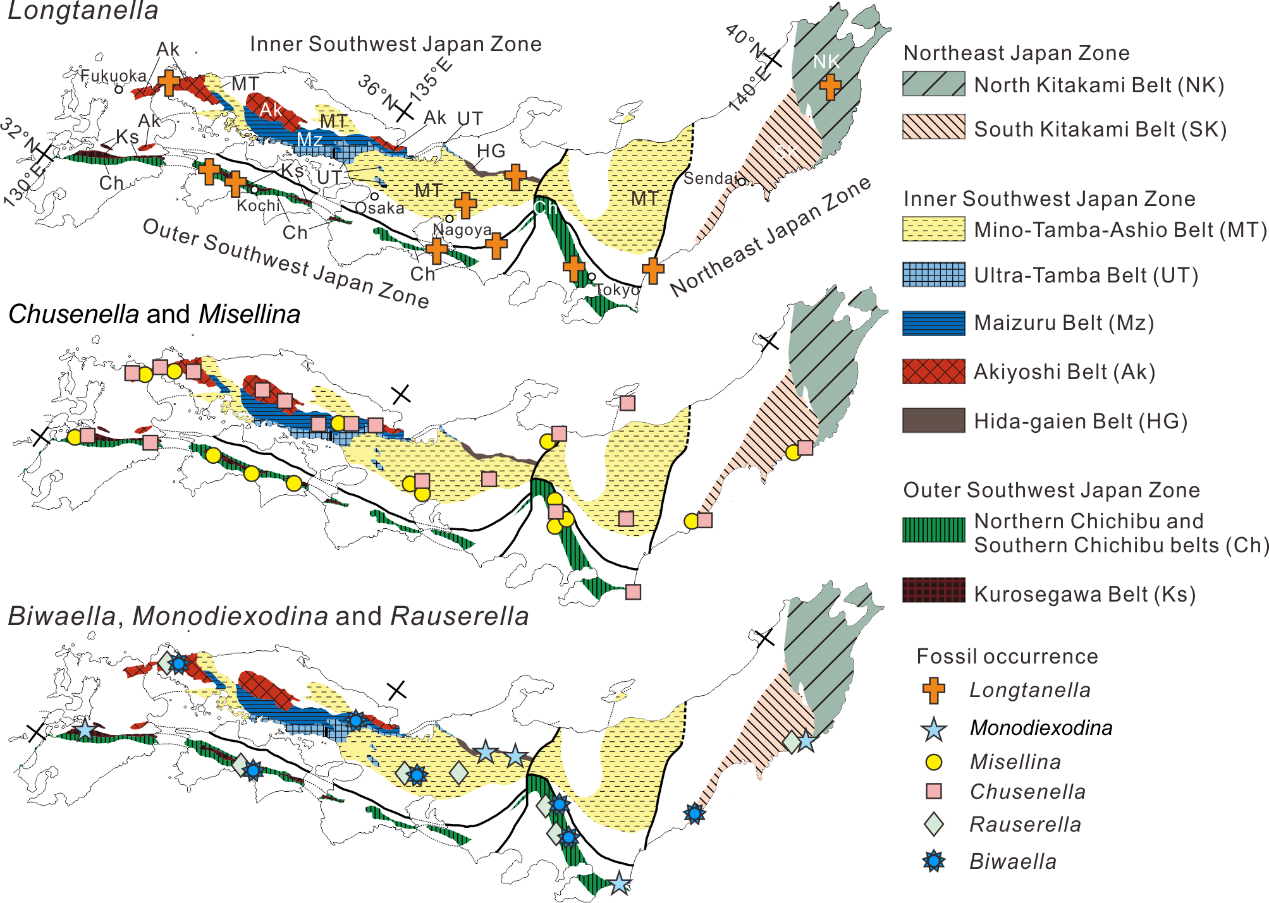


**Figure S6.** Occurrences of the selected fusulinacean genera and *Longtanella* in Japanese Islands (Tectonic map revised after Wallis *et al.* 2020). T.I. created this figure using Adobe Illustrator CC 2017 (https://helpx.adobe.com/au/illustrator/release-note/illustrator-cc-2017-21-0-release-notes.html).


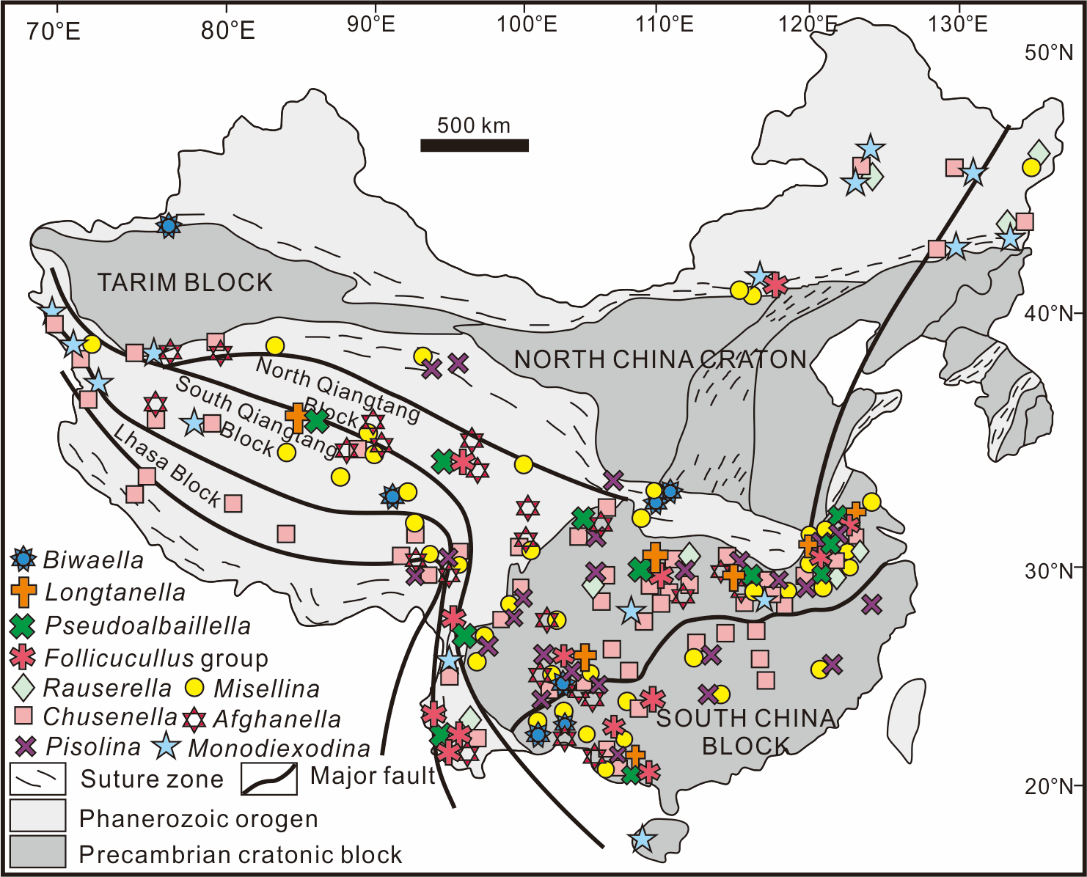


**Figure S7.** Occurrences of the selected fusulinacean and radiolarian genera in Mainland China (Tectonic map revised after Zheng *et al.* 2013). Y.F.X. created this figure using CorelDRAW X4.


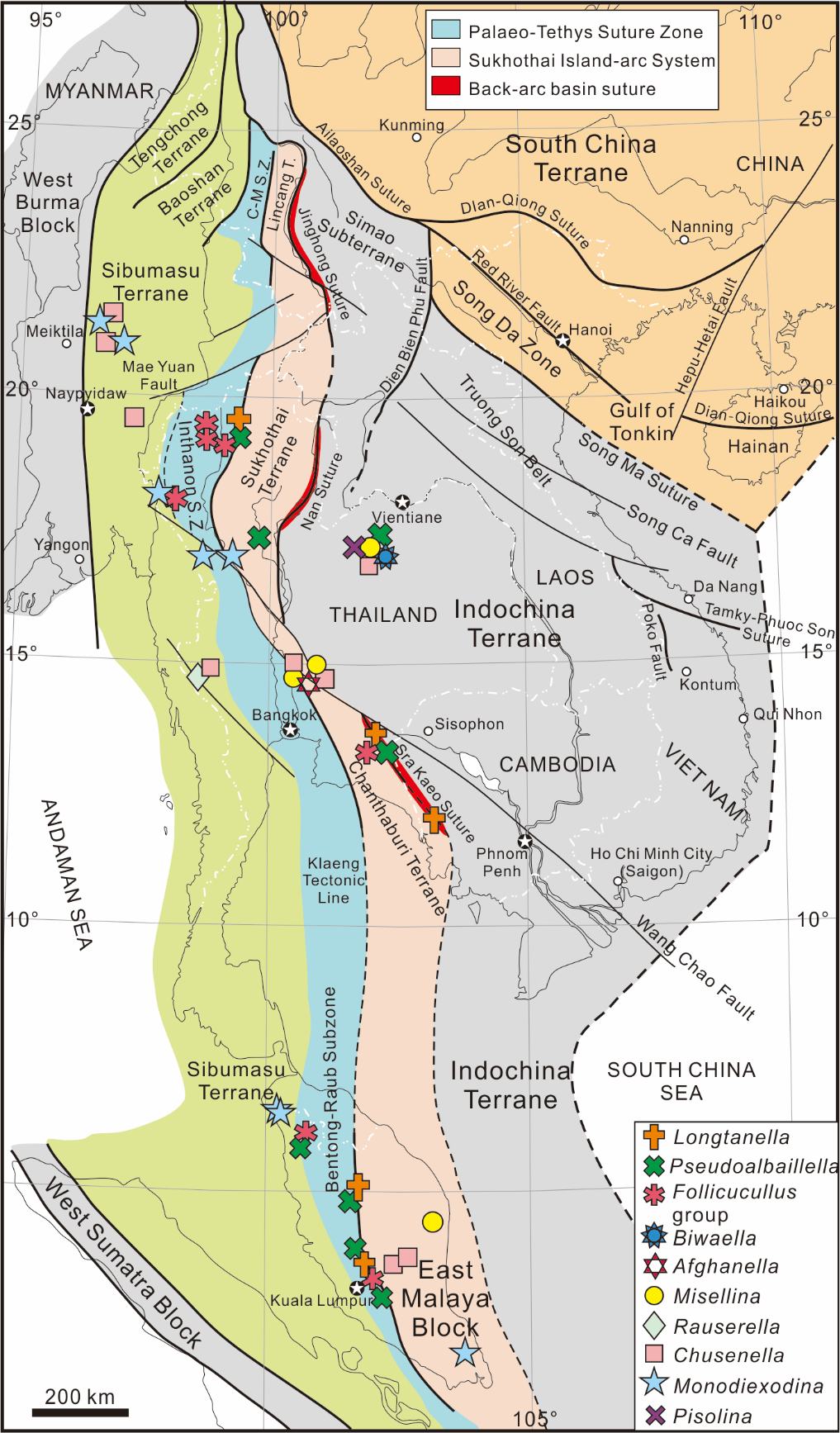


**Figure S8.** Occurrences of the selected fusulinacean and radiolarian genera in Sundaland (Tectonic map revised after Metcalfe 2013). Y.F.X. created this figure using CorelDRAW X4.

**Supplement 2. UAZ range (age) of each sample in the Shiti section decided by radiolarian species.**


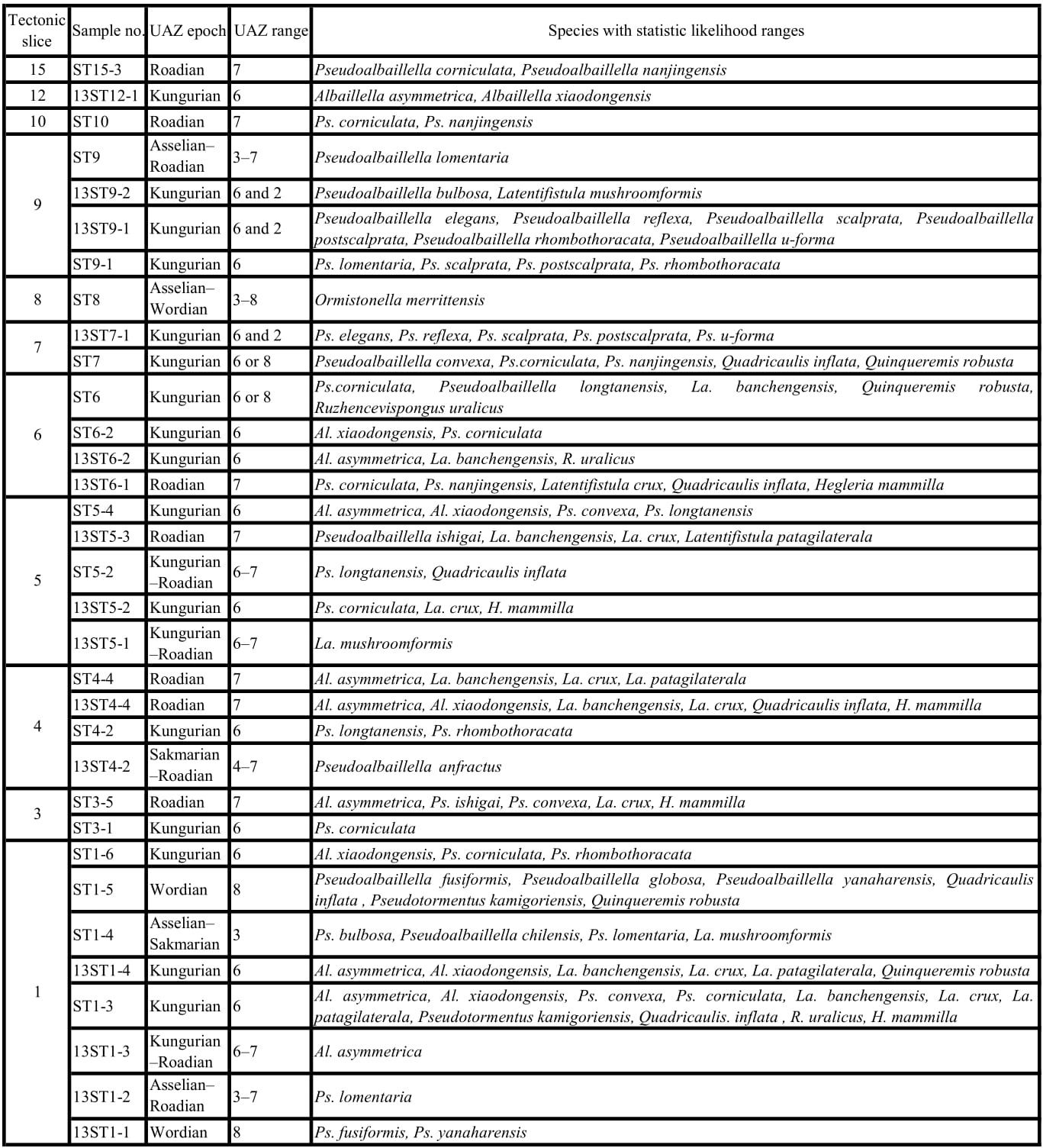


Abbreviations: *Ps*., *Pseudoalbaillella*; *Al*., *Albaillella*; *R.*, *Ruzhencevispongus*; *La*., *Latentifistula*; *H.*, *Hegleria*.

**Supplement 3. Occurrences of fusulinaceans and radiolarians**

| Genus | Region | Tectonic division | References |
| --- | --- | --- | --- |
| *Monodiexodina* | Japan Islands | Hida-gaien | Niwa et al., 2004 |
|  |  |  | Ueno and Tazawa, 2004 |
|  |  | Kurosegawa belts | Kanmera, 1963 |
|  |  |  | Tazawa and Hasegawa, 2007 |
|  | Mainland China (exclusive of the Changning-Menglian Suture Zone) | Heilongjiang province | Han, 1980 |
|  |  |  | Han, 1981 |
|  |  | Jilin province | Han, 1980 |
|  |  | Nei Mongol | Han, 1976 |
|  |  | Tibet province | Wang et al., 1982a |
|  |  |  | Nie and Song, 1983a |
|  |  |  | Geng et al., 2012 |
|  |  |  | Zhang et al. 2014b |
|  |  |  | Zhang et al. 2013 |
|  |  | Hainan province | Sheng, 1965 |
|  |  | Hubei province | Chen, 1984 |
|  |  | Jiangsu province province | Zhou and Zhang, 1984 |
|  |  | Sichuan province | Chen and Yang, 1978 |
|  | Sundaland and the Changning-Menglian Suture Zone | Ailaoshan Suture | Igo et al., 1979 |
|  |  | Inthanon Suture Zone | Basir Jasin, 1991 |
| *Rauserella* | Japan Islands | Akiyoshi Belt | Kawano, 1961 |
|  |  |  | Kobayashi, 2010 |
|  |  |  | Kobayashi, 2012a |
|  |  |  | Kobayashi, 2012b |
|  |  |  | Kobayashi, 2013 |
|  |  | Mino-Tamba-Ashio Belt | Kobayashi, 2006a |
|  |  |  | Kobayashi, 2011a |
|  |  |  | Ueno et al., 2006 |
|  |  |  | Kobayashi and Furutani, 2018 |
|  |  | Northern and Southern Chichibu belts | Morikawa, 1956 |
|  |  |  | Kobayashi, 1988a |
|  |  |  | Kobayashi, 2006b |
|  |  |  | Kobayashi, 2007 |
|  |  |  | Kobayashi, 2016 |
|  | Mainland China (exclusive of the Changning-Menglian Suture Zone) | Anhui province | Wang et al., 1982b |
|  |  | Guizhou province | Yang, 1985 |
|  |  | Jiangsu province province | Wang et al., 1982b |
|  |  | Jilin province | Han, 1980 |
|  |  | Sichuan province | Zhang and Wang, 1974 |
|  |  |  | Chen and Yang, 1978 |
|  | Sundaland and the Changning-Menglian Suture Zone | Thailand | Ueno, 2003 |
|  |  | Yunnan province | Zhou et al., 1987 |
|  |  |  | Zhou, 2001 |
|  |  |  | Shi et al., 2008 |
| *Afghanella* | Japan Islands | Akiyoshi Belt | Hanzawa, 1954 |
|  |  |  | Kanmera, 1957 |
|  |  |  | Toriyama, 1958 |
|  |  |  | Kawano, 1961 |
|  |  |  | Hanzawa and Murata, 1963 |
|  |  |  | Kobayashi, 1988b |
|  |  |  | Kobayashi, 2012a |
|  |  |  | Kobayashi, 2012b |
|  |  |  | Ishii, 1990 |
|  |  |  | Ozawa and Kobayashi, 1990 |
|  |  |  | Ueno, 1992 |
|  | Mainland China (exclusive of the Changning-Menglian Suture Zone) | Guangxi province | Lin et al., 1977 |
|  |  |  | Li, 1989 |
|  |  | Guizhou province | Sheng, 1963 |
|  |  |  | Liu et al., 1978 |
|  |  |  | Yang, 1985 |
|  |  |  | Chang et al., 2012 |
|  |  | Hubei province | Chen, 1984 |
|  |  | Hunan province | Lin et al, 1977 |
|  |  |  | Xie, 1982 |
|  |  | Sichuan province | Chen and Yang, 1978 |
|  |  |  | Zhang,1982 |
|  |  |  | Sheng, 1963 |
|  |  | Qinghai province | Sheng, 1958 |
|  |  |  | Sheng and Sun, 1975 |
|  |  |  | Niu and Wu, 2016 |
|  |  | Tibet province | Zhang, 1982 |
|  |  |  | Cheng et al., 2005 |
|  |  | Xinjiang province | Da and Su, 1983 |
|  |  |  | Da, 1985 |
|  |  |  | Sun and Zhang, 1985 |
|  |  |  | Sun, 1993 |
|  | Sundaland and the Changning-Menglian Suture Zone | Yunnan province | Zhou et a., 1987 |
|  |  |  | Zhou, 2001 |
|  |  |  | Shi et al., 2008 |
|  |  | Thailand | Toriyama and Kanmera, 1979 |
|  |  |  | Dawson, 1993 |
| *Biwaella* | Japan Islands | Akiyoshi Belt | Kobayashi 2012b |
|  |  |  | Kobayashi 2017 |
|  |  | Mino-Tamba-Ashio | Morikawa and Isomi, 1960 |
|  |  |  | Morikawa and Isomi, 1961 |
|  |  |  | Kobayashi and Furutani, 2009 |
|  |  |  | Kobayashi and Furutani, 2019 |
|  |  | Northern and Southern Chichibu belts | Morikawa and Kobayashi, 1960 |
|  |  |  | Ishizaki, 1962b |
|  |  |  | Suyari, 1962 |
|  |  |  | Kobayashi, 1993 |
|  |  |  | Kobayashi, 2005a |
|  |  |  | Kobayashi, 2005b |
|  | Mainland China (exclusive of the Changning-Menglian Suture Zone) | Guangxi province | Huang and Zeng, 1984 |
|  |  | Guizhou province | Liu et al., 1978 |
|  |  |  | Xia, 1994 |
|  |  | Shaanxi province | Wang and Sun, 1973 |
|  |  |  | Ding et al., 1987 |
|  |  |  | Ding et al., 1991 |
|  |  | Xinjiang province | Da and Su, 1983 |
|  | Sundaland and the Changning-Menglian Suture Zone | Thailand | Sakagami and Iwai, 1974 |
|  |  | Changning-Menglian suture zone | Sakagami and Iwai, 1974 |
| *Misellina* | Japan Islands | Hida-gaien Belt | Niwa et al., 2004 |
|  |  | Kurosegawa Belt | Toriyama, 1947 |
|  |  |  | Kanmera, 1956 |
|  |  |  | Kanmera, 1963 |
|  |  | Akiyoshi Belt | Toriyama, 1958 |
|  |  |  | Kobayashi, 1977 |
|  |  |  | Ozawa and Kobayashi, 1990 |
|  |  |  | Ueno, 1991 |
|  |  | Mino-Tamba-Ashio Belt | Morikawa and Isomi, 1961 |
|  |  |  | Kobayashi, 2008 |
|  |  | Northern and Southern Chichibu belts | Fujimoto, 1936 |
|  |  |  | Ishizaki, 1962a |
|  |  |  | Ishizaki, 1963 |
|  |  |  | Suyari, 1962 |
|  |  |  | Takaoka, 1966 |
|  |  |  | Kobayashi, 1977 |
|  |  |  | Kobayashi, 2005a |
|  | Sundaland and the Changning-Menglian Suture Zone | Yunnan province | Zhou et a., 1987 |
|  |  |  | Zhou, 2001 |
|  |  |  | Shi et al., 2008 |
| *Chusenella* | Japan Islands | Akiyoshi Belt | Sada and Yokoyama, 1966 |
|  |  |  | Kobayashi, 1988b |
|  |  |  | Kobayashi, 2010 |
|  |  |  | Kobayashi, 2012a |
|  |  |  | Kobayashi, 2012b |
|  |  |  | Ozawa and Kobayashi, 1990 |
|  |  |  | Davydov and Schmitz, 2019 |
|  |  | Kurosegawa Belt | Kanmera, 1954 |
|  |  |  | Kobayashi, 2001 |
|  |  |  | Tazawa and Hasegawa, 2007 |
|  |  | Mino-Tamba-Ashio Belt | Kobayashi, 2006a |
|  |  |  | Kobayashi, 2011a |
|  |  |  | Kobayashi and Furutani, 2018 |
|  |  | Southern Chichibu belts | Kobayashi, 2005a |
|  |  |  | Kobayashi, 2006b |
|  |  |  | Kobayashi, 2007 |
|  |  |  | Kobayashi, 2011b |
|  | Mainland China (exclusive of the Changning-Menglian Suture Zone) | Anhui province | Wang et al., 1982b |
|  |  | Guangxi province | Hsu, 1942 |
|  |  |  | Sheng, 1963 |
|  |  |  | Lin et al., 1977 |
|  |  |  | Li, 1989 |
|  |  | Guizhou province | Liu et al., 1978 |
|  |  |  | Yang, 1985 |
|  |  | Hubei province | Chen, 1956 |
|  |  |  | Lin et al., 1977 |
|  |  |  | Lin, 1984 |
|  |  | Hunan province | Chen, 1956 |
|  |  |  | Lin et al., 1977 |
|  |  |  | Xie, 1982 |
|  |  |  | Cao et al., 2013 |
|  |  | Jiangsu province province | Wang et al., 1982b |
|  |  | Jiangxi province | Wang et al., 1982b |
|  |  | Sichuan province | Zhang and Wang, 1974 |
|  |  |  | Chen and Yang, 1978 |
|  |  |  | Zhang, 1982 |
|  |  | Shaanxi province | Sheng, 1956 |
|  |  | Qinghai province | Niu and Wu, 2016 |
|  |  | Xinjiang province | Da and Su, 1983 |
|  |  |  | Sun and Zhang, 1988 |
|  |  |  | Gaetani and Leven, 2014 |
|  |  | Tibet province | Chu, 1982 |
|  |  |  | Wang et al., 1982a |
|  |  |  | Zhang, 1982 |
|  |  |  | Nie and Song, 1983b |
|  |  |  | Wang and Zhou, 1986 |
|  |  |  | Cheng et al., 2005 |
|  |  |  | Zhang et al., 2009 |
|  |  |  | Zhang et al., 2013 |
|  |  |  | Zhang et al., 2014 |
|  |  |  | Zhang et al., 2019 |
|  |  | Jilin province | Han, 1980 |
|  | Sundaland and the Changning-Menglian Suture Zone | Yunnan province | Zhou, 2001 |
|  |  |  | Shi et al., 2005 |
|  |  |  | Shi et al., 2008 |
|  |  |  | Shi et al., 2017 |
|  |  |  | Huang et al., 2009 |
|  |  |  | Huang et al., 2015 |
|  |  |  | Huang et al., 2017 |
|  |  | Myanmar | Zhang et al., 2020 |
|  |  | Thailand | Pitakpaivan, 1966 |
|  |  |  | Sakagami and Iwai, 1974 |
|  |  |  | Toriyama, 1976 |
|  |  |  | Toriyama and Kanmera, 1977 |
|  |  |  | Toriyama and Kanmera, 1979 |
|  |  |  | Dawson, 1993 |
|  |  |  | Ueno, 2003 |
| *Pisolina* | Mainland China (exclusive of the Changning-Menglian Suture Zone) | Anhui province | Regional Geological Survey Team of Anhui province, 1982 |
|  |  |  | Wang et al., 1982b |
|  |  | Fujian province | Hong et al., 1986 |
|  |  | Guangxi province | Lin et al., 1977 |
|  |  |  | Li, 1989 |
|  |  | Guizhou province | Liu et al., 1978 |
|  |  |  | Yang, 1985 |
|  |  |  | Zhang et al., 2010 |
|  |  | Hubei province | Lin et al., 1977 |
|  |  |  | Chen, 1984 |
|  |  |  | Lin, 1984 |
|  |  | Hunan province | Lin et al., 1979 |
|  |  |  | Zhang and Hong, 2000 |
|  |  | Jiangsu province province | Zhou and Zhang, 1984 |
|  |  | Qinghai province | Sheng and Sun, 1975 |
|  |  | Shaanxi province | Sheng, 1956 |
|  |  | Sichuan province | Zhang and Wang, 1974 |
|  |  |  | Chen and Yang, 1978 |
|  |  |  | Zhang, 1982 |
|  |  | Zhejiang province | Wang and Tang, 1986 |
|  | Sundaland and the Changning-Menglian Suture Zone | Indochina Terrane | Ueno and Sakagami, 1993 |
| *Longtanella* | Japan Islands | Northern and Southern Chichibu belt | Hori, 2004 |
|  |  |  | Ujiie and Oba, 1991 |
|  |  |  | Sashida, 1995 |
|  |  |  | Suyari et al., 1982 |
|  |  | Kurosegawa Belt | Hada et al., 1992 |
|  |  |  | Kashiwagi and Isaji, 2015 |
|  |  | North Kitakami Belt | Kametaka et al., 2005 |
|  |  | Hida-gaien Belt | Niko et al., 1987 |
|  |  | Nagato Tectonic Zone | Isozaki and Tamura, 1989 |
|  |  | Mino Terrane | Ito et al., 2016 |
|  | Far east Russia | Sikhote-Alin | Rudenko and Panasenko, 1997 |
|  | British Columbia |  | Harms and Murchey, 1992 |
|  | Mainland China (exclusive of the Changning-Menglian Suture Zone) | Qinghai province | Li and Bian, 1993 |
|  |  | Guangxi province | Yao et al., 2004 |
|  |  |  | Shimakawa and Yao, 2006 |
|  |  |  | Wu et al., 1994 |
|  |  | Guizhou province | Yu, 1996 |
|  |  | Jiangsu province province | Sheng and Wang, 1985 |
|  |  | Hubei province | Shi et al., 2016 |
|  |  | Anhui province | Nagai et al., 1998 |
|  |  |  | Kametaka et al., 2009 |
|  |  |  | Ito et al., 2013 |
|  |  |  | Wang, 1993a |
|  | Sundaland and the Changning-Menglian Suture Zone | Yunnan province | Feng, 1992 |
|  |  | Bentong-Raub suture zone | Basir Jasin and Che Ali, 1997 |
|  |  |  | Metacalfe et al., 1999 |
|  |  | Sra Kaeo Suture Zone | Saesaengseerung et al., 2009 |
|  |  |  | Udchachon et al., 2018 |
|  |  | Inthanon Suture Zone | Sashida et al., 1993 |

**Reference list**

Basir Jasin, 1991, Significance of *Monodiexodina* (Fusulininacea) in geology of Peninsular Malaysia. Bulletin of the Geological Society of Malaysia 29, 171–181.

Basir Jasin & Che Aziz, A. 1997. Lower Permian Radiolaria from the Pos Blau area, Ulu Kelantan, Malaysia. Journal of Asian Earth Sciences, 15, 327–339.

Cao, C.Q., Zhang, M.Y., Zheng, Q.F., Yuan, D.X., Chen, J. and Ding, Y., 2013. The Permian Capitanian stratigraphy at the Rencunping section, Sangzhi County of Hunan province and its environmental implications. Journal of Stratigraphy, 37, 485–498. (in Chinese with English abstract)

Chang, X.L., Shi, H., Li, K. and Hu, F., 2012. Biostratigraphy and chronostratigraphic boundaries of the Permian at the Ganzhai section, Pingtang, Guizhou province. Acta Micropalaeontologica Sinica, 29, 391–401. (in Chinese with English abstract)

Chen, S., 1956, The Fusulinidae of South China, Part II. Fusulinidae of the Maokou Limestone in the southwestern provinces of China. Palaeontologica Sinica, 140 (New Series 6), 1–71. (in Chinese with English abridged parts)

Chen, G.X., 1984, Fusulinida. p. 12–73, 691–703. In Regional Geological Surveying Team of Hubei province (ed.) The Palaeontological Atlas of Hubei province Province. Hubei province Science and Technology Press, Wuhan. (in Chinese).

Chen, J.R. and Yang, C.R., 1978. Fusulinida. In Institute of Geological Sciences of Southwest China (ed.) Palaeontological Atlas of Southwest China. Sichuan province (2) Carboniferous to Mesozoic. Geological Publishing House, Beijing, p. 17–123. (in Chinese, title translated)

Cheng, L.R., Li, C., Zhang, Y.C. and Wu, S.Z., 2005, The *Polydiexodina* (fusulinids) fauna from central Qiangtang, Tibet province, China. Acta Micropalaeontologica Sinica, 22, 152–162. (in Chinese with English abstract).

Chu, S.F., 1982, Lower Permian fusulinids from Xainza County, Xizang (Tibet province). Contribution to the Geology of the Qinghai province-Xizang (Tibet province), 7, 110–136. (in Chinese with English abstract)

Da, Y.T., 1985, An occurrence of *Eopolydiexodina* fauna (fusulinids) from lower Permian of Mt. Kunlun-Altun. Acta Micropalaeontologica Sinica 2, 387–394. (in Chinese with English abstract)

Da, Y.T. and Su, Q.L., 1983, Fusulinida. In Geological Survey Team of the Xinjiang province Geological Bureau, Institute of Geological Science oft he Xinjiang province Geological Bureau and the Geological Survey Branch of Xijiang Oil Bureau (eds.) The Paleontological Atlas of Northwest China. Volume of Xinjiang province Uyghurs Automonous Region. (2) Late Paleozoic. Geological Publshing House, Beijing, pp. 7–113. (in Chinese, title translated)

Davydov, V.I. and Schmitz, M.D., 2019. High-precision radioisotopic ages for the lower Midian (upper Wordian) Stage of the Tethyan time scale, Shigeyasu Quarry, Yamaguchi Prefecture, Japan. Palaeogeography, Palaeoclimatology, Palaeoecology, 527, 133–145.

Dawson, O., 1993, Fusuline foraminiferal biostratigraphy and carbonate facies of the Permian Ratburi Limestone, Saraburi, central Thailand. Journal of Micropalaeontology, 12, 9–33.

Ding, P.Z., Jin, T.A and Sun, X.F., 1987, An excursion guide to Permian geology of Xikou area, Zhen’an County, Shaanxi. Bulletin of the Xi’an Institute of Geology and Mineral Resources, Chinese Academy of Geological Science, 15, 113–138. (In Chinese with English abstract)

Ding, P.Z., Xia, G.Y. Li, L., Yu, X.G., Zhao, S.Y. and Zhao, Z., 1991, The Carboniferous-Permian boundary and faunas from Xikou area, Zhen’an, Shaanxi in eastern Qinling Range. Bulletin of the Tianjin Institute, Geology and Mineral Resources Research, 24, 1–202. (in Chinese with English abridged parts)

Feng, Q. L. 1992. Permian and Triassic radiolarian biostratigraphy in south and southwest China. Earth Science-Journal of China University of Geosciences, 31, 51–62.

Fujimoto, H., 1936, Stratigraphical and palaeontological studies of the Titibu System of the Kwanto-Mountainland. Part 2. Palaeontology. Science Reports on the Tokyo Bunrika Daigaku, Section C, 1, 29–125.

Gaetani, M. and Leven, E.Ja., 2014, The Permian succession of the Shaksgam Valley, Sinkiang (China). Italian Journal of Geosciences 133, 45–62.

Geng, Q.R., Peng, Z.M. and Zhang, Z., 2012, New advances in the study of Carboniferous-Permian paleontology in Guoganjianianshan-Rongma area of Qiangtang region, Tibet provincean Plateau. Geological Bulletin of China, 31, 510– 520. (In Chinese with English abstract)

Hada, S., Salo, E., Takeshima, H. & Kawakami, A. 1992. Age of the covering strata in the Kurosegawa Terrane: dismembered continental fragment in southwest Japan. Palaeogeography, Palaeoclimatology, Palaeoecology, 96, 59–69.

Han, J.X., 1976. Fusulinida. In Geological Bureau of Inner Mongol Autonomous Region and Institute of the Geological Science of the Northeast China (eds.) Paleontological Atlas of Northeast China. Inner Mongol Volume. (1) Paleozoic. Geological Publishing House, Beijing, pp. 23–63. (in Chinese, title translated).

Han, J.X., 1980. Fusulinida. In Shenyang Institute of Geology and Mineral Resources (ed.) Paleontological Atlas of Northeast China. (1) Paleozoic Volume. Geological Publishing House, Beijing, pp. 18–95. (in Chinese).

Han, J.X., 1981, The morphology, evolution and distribution of the genus Monodiexodina and its allied genera. Bulletin of the Shenyang Institute of Geology and Mineral Resources, Chinese Academy of Geological Sciences, 1, 90–102. (in Chinese with English abstract)

Hanzawa, S., 1954, Notes on Afghanella and Sumatrina from Japan. Japanese Journal of Geology and Geography, 24, 1–14.

Hanzawa, S. and Murata, M., 1963, The paleontologic and stratigraphic considerations on the Neoschwagerninae and Verbeekininae, with the descriptions of some fusulinid foraminifera from the Kitakami Massif, Japan. Science Reports of the Tohoku University. 2nd Series, Geology, 35, 1–31.

Harms, T. A. & Murchey, B. L. 1992. Setting and occurrence of Late Paleozoic radiolarians in the Sylvester allochthon, part of a proto-Pacific ocean floor terrane in the Canadian Cordillera. Palaeogeography, Palaeoclimatology, Palaeoecology, 96, 127–139.

Hong, Z.Y., Jiang, J.C. and Liu, Q.P., 1986, Early Permian Chihsia Formation fusulinid faunas from Gaodi of Fukou, Shaxian County, Fujian province Province. Geology of Fujian province 1986(4), 45–64 with plates 1 to 3 (in Chinese).

Hori, N., 2004. Permian radiolarians from chert of the Chichibu Belt in the Toyohashi district, Aichi Prefecture, Southwest Japan. Bulletin of the Geological Survey of Japan 55, 287–301. (in Japanese with English abstract)

Hsu, Y.C., 1942, On the type species of Chusenella. Bulletin of the Geological Society of China, 22, 175–176.

Huang, Z.X. and Zeng, X.L., 1984, The early Early Permian (Longlinian Stage) fusulinid fauna from Longlin, Guangxi province. Earth Science–Journal of Wuhan College of Geology 3, 11–24. (in Chinese with English abstract)

Huang, H., Jin, X.C., Shi, Y.K. and Yang, X.N., 2009, Middle Permian western Tethyan fusulinids from southern Baoshan Block, western Yunnan province, China. Journal of Paleontology, 83, 880–896.

Huang, H., Shi, Y.K. and Jin, X.C., 2015. Permian fusulinid biostratigraphy of the Baoshan Block in western Yunnan province, China with constraints on paleogeography and paleoclimate. Journal of Asian Earth Sciences, 104, 127–144.

Huang, H., Shi, Y.K. and Jin, X.C., 2017. Permian (Guadalupian) fusulinids of Bawei Section in Baoshan Block, western Yunnan province, China: Biostratigraphy, facies distribution and paleogeographic discussion. Palaeoworld, 26, 95–114.

Igo, Hy., Rajah, S.S. and Kobayashi, F., 1979, Permian fusulinaceans from the Sungei Sedili area, Johre, Malaysia. p. 95–118. In Kobayashi, T., Toriyama, R. and Hashimoto, W. (eds.) Geology and Palaeontology of Southeast Asia. Volume 20. University of Tokyo Press, Tokyo.

Ishii, K., 1990, Provinciality of some fusulinacean faunas of Japan. In Ichikawa, K., Mizutani, S., Hara, I., Hada, S. and Yao, A. (eds.) Pre-Cretaceous Terranes of Japan. p. 297–305. Osaka City University, Osaka.

Ishizaki, K., 1962a, Stratigraphical and paleontological studies of the Onogahara and its neighboring area, Kochi and Ehime prefectures, southwest Japan. Science Reports of the Tohoku University. 2nd Series (Geology), 34, 95 – 185.

Ishizaki, K., 1962b, A new locality and faunal assemblage of fusulinids from the limestone in the area west of Ryoseki, Kochi Prefecture. Science Reports of the Tohoku University. 2nd Series (Geology), Special Volume, 5, 107–118.

Ishizaki, K., 1963, Verbeekininae from the inferred upper Wolfcampian limestone in the west of Ryoseki, Kochi Prefecture. Transactions and Proceedings of the Palaeontological Society of Japan, New Series, 50, 51–64.

Isozaki, Y. & Tamura, H. 1989. Late Carboniferous and Early Permian radiolarians from the Nagato Tectonic Zone and their implication to geologic structure of the Inner Zone, Southwest Japan. The Memoirs of Geological Society of Japan, 33, 167–176. [In Japanese with English abstract.]

Ito, T., Feng, Q. L. & Matsuoka, A. 2013. Radiolarian faunal change in the Middle Permian Gufeng Formation in the Liuhuang section, Chaohu, South China. Science Reports of Niigata University (Geology), 28, 39–49.

Ito, T., Kitagawa, Y. & Matsuoka, A. 2016. Middle and Late Permian radiolarians from chert blocks within conglomerates of the Kamiaso Unit of the Mino Terrane in Gifu Prefecture, central Japan. Journal of the Geological Society of Japan, 122, 249–259. [In Japanese with English abstract.]

Kametaka, M., Nakae, S. & Kamada, K. 2005. Early Permian radiolarians from siliceous mudstone in the Rikuchu-Seki District, North Kitakami Terrane. Bulletin of the Geological Survey of Japan, 56, 237–243. [In Japanese with English abstract.]

Kametaka, M., Nagai, H., Zhu, S. Z. & Takebe, M. 2009. Middle Permian radiolarians from Anmenkou, Chaohu, Northeastern Yangtze platform, China. Island Arc, 18, 108–125.

Kanmera, K., 1954, Fusulinids from the upper Permian Kuma Formation, southern Kyushu, Japan. With special reference to the fusulinid zone in the upper Permian of Japan. Memoirs of the Faculty of Science, Kyushu University, Series D, Geology, 4, 1–38.

Kanmera, K., 1956, *Toriyamaia*, a new Permian fusulinid genus from the Kuma Massif, Kyushu, Japan. Transactions and Proceedings of the Palaeontological Society of Japan, New Series, 21, 251–257.

Kanmera, K., 1957, Revised classification of *Cancellina* and *Neoschwagerina*, and evolution of Sumatrininae and Neoschwagerininae. Memoirs of the Faculty of Science, Kyushu University, Series D, Geology, 6, 47–64.

Kanmera, K., 1963, Fusulines of the middle Permian Kozaki Formation of southern Kyushu. Memoirs of the Faculty of Science, Kyushu University, Series D, Geology, 14, 79–141.

Kashiwagi, K. & Isaji, S. 2015. Paleozoic and Mesozoic radiolarians from chert pebbles and cobbles of the Lower Cretaceous Choshi Group, Japan. Natural History Research (Natural History Museum and Institute, Chiba), 13, 35–46.

Kawano, M., 1961, Stratigraphical and paleontological studies of the Paleozoic formations in the western part of the Chugoku Massif. Bulletin of the Faculty of Education, Yamaguchi University, Mathematics & Science, 1–133.

Kobayashi, F., 1977, Some considerations on the ancestor of the family Verbeekinidae (Fusulinacea). Transactions and Proceedings of the Palaeontological Society of Japan, New Series, 105, 1–16.

Kobayashi, F., 1988a. Late Paleozoic foraminifers of the Ogawadani Formation, southern Kwanto Mountains, Japan. Transactions and Proceedings of the Palaeontological Society of Japan, New Series, 150, 435–452.

Kobayashi, F., 1988b. Middle Permian foraminifers of the Omi Limestone, central Japan. Bulletin of the Nature and Science Museum of Tokyo, Series C, 14, 1 1–35.

Kobayashi, F., 1993, Fusulinaceans contained in pebbles of the infraformational conglomerate of the Kanyo Formation, north of Itsukaichi, southern Kwanto Mountains, Japan. Human and Nature, 2, 125–137.

Kobayashi, F., 2001, Faunal analysis of Permian foraminifers of the Kuma Formation in the Kurosegawa Belt of west Kyushu, southwest Japan. News of Osaka Micropaleontologistis (NOM), Special Volume, 12, 61–84.

Kobayashi, F., 2005a, Permian foraminifers from the Itsukaichi-Ome area, west Tokyo, Japan. Journal of Paleontology, 79, 413–432.

Kobayashi, F., 2005b. Early Permian fusulinaceans in the Hanagiri-Shimokuzu area, eastern part of the Kanto Mountains, Japan. Human and Nature, 9, 11–31.

Kobayashi, F., 2006a, Middle Permian foraminifers of the Izuru and Nabeyama formations in the Kuzu area, Tochigi Prefecture, Japan Part 1. Schwagerinid, neoschwagerinid, and verbeekinid fusulinoideans. Paleontological Research, 10, 37–59.

Kobayashi, F., 2006b, Middle Permian foraminifers of Kaize, southern part of the Saku Basin, Nagano Prefecture, central Japan. Paleontological Research, 10, 179–194.

Kobayashi, F., 2007, Foraminiferal fauna and lithofacies of middle Permian limestone blocks in the middle course of Kuma River (Osakama), southern margin of the Chichibu Terrane in west Kyushu, Japan. Paleontological Research, 11, 337–347.

Kobayashi, F., 2008, Late early Permian (Kungurian) fusulines from Kamiishizu, south of Sekigahara, Gifu Prefecture, Japan. Humans and Nature, 19, 27–33.

Kobayashi, F., 2010, Late middle Permian (Capitanian) foraminifers from the uppermost part of the Taishaku Limestone, Akiyoshi Terrane, Japan. Paleontological Research, 14, 260–276.

Kobayashi, F., 2011a, Permian fusuline faunas and biostratigraphy of the Akasaka Limestone (Japan). Revue de Paléobiologie, Genève, 30, 431–574.

Kobayashi, F., 2011b, Middle Permian (Wordian) foraminifers of the Furen Limestone, Oita Prefecture, Japan. Humans and Nature, 22, 21–25.

Kobayashi, F., 2012a, Comparative study of the Capitanian (late Guadalupian) foraminiferal faunas in the *Lepidolina shiraiwaensis* Zone of the Akiyoshi Limestone Group, SW Japan. Journal of Foraminiferal Research, 42, 82–101.

Kobayashi, F., 2012b, Late Paleozoic foraminifers from limestone blocks and fragments of the Permian Tsunemori Formation and their connection to the Akiyoshi Limestone Group, Southwest Japan. Paleontological Research 16, 219–243.

Kobayashi, F., 2013. Late Permian (Lopingian) foraminifers from the Tsukumi Limestone, Southern Chichibu Terrane of Eastern Kyushu, Japan. The Journal of Foraminiferal Research. 43(2): 154–169.

Kobayashi, F., 2016, Morphologic variation and microspheric forms of Parafusulina japonica from Tamanouchi, Itsukaichi-Ome area, west Tokyo, Japan. Paleontological Research 20, 394–406.

Kobayashi, F., 2017, Late Carboniferous and early Permian fusulines of the Akiyoshi Limestone Group in the Wakatakeyama area, Akiyoshi (Japan) –Biostratigraphy, biogeography, and biodiversity. Revue de Paléobiologie, Genève, 36, 1–155.

Kobayashi, F. and Furutani, H., 2009, Early Permian fusulines from the western part of Mt. Ryozen, Shiga Prefecture, Japan. Humans and Nature 20, 29–54.

Kobayashi, F. and Furutani, H., 2018. Reconsideration of the Late Carboniferous and Permian fusuline faunas in the Sakamototoge area (northern part of the Mino Belt), Gifu Prefecture, Japan. Paleontological Research 22, 373–389.

Kobayashi, F. and Furutani, H., 2019, Late early Permian fusulines along Gongendani, south of Mt. Ryozen, Shiga Prefecture, central Japan. Paleontological Research 23, 131–151.

Li, J.L. (ed.), 1989, Guangxi province Fusulinids. 213 pp.. Guangxi province Normal University Publishing, Guilin. (in Chinese).

Li, H. S. & Bian, Q. T. 1993. Upper Paleozoic Radiolaria of the Xijin Ulan -Gangqiqu Ophiolite Complex, Kekexili. Geoscience-Journal of Graduate School, China University of Geosciences, 7, 410–420. [In Chinese with English abstract.]

Lin, J.X., 1984, Fusulinida. p. 151–177, 318–322, 330, 375–382. In Yichang Institute of Geology and Mineral Resources (ed.) Biostratigraphy of the Yangtze Gorge Area. (3) Late Palaeozoic Era. Geological Publishing House, Beijing. (in Chinese).

Lin J.X., Li, J.L., Chen, G.X., Zhou, Z.R., Zhang, B.F., 1977. Order Fusulinacea. p. 4–97, 744–762. In Institute of Geological Science in Hubei province Province (ed.) Paleontological Atlas of Middle West China. Geological Publishing House, Beijing. (in Chinese, title translated)

Lin, J.X., Pan, S.S. and Meng, F.Y., 1979, Late Carboniferous and early lower Permian fusulinids from Jiahe, Huna. Acta Palaeontologica Sinica, 18, 561–572. (in Chinese with English abstract)

Liu, C.A., Xiao, X.M., Dong, W.L., 1978, Fusulinida. p. 12–98, 550–638. In Regional Geological and Paleontological Survey Team of Guizhou province (ed.) Paleontological Atlas of Southwest China. Guizhou province Part. (2) Carboniferous–Quaternary. Geological Publishing House, Beijing. (in Chinese, title translated).

Metcalfe, I., Spiller, F. C. P., Liu, B. P., Wu, H. R. & Sashida, K. 1999. The Palaeo-Tethys in Mainland East and Southeast Asia: contributions from radiolarian studies. Pp. 259–281 in I. Metcalfe (ed.) Gondwana Dispersion and Asian Accretion. IGCP321 Final Results Volume. A.A. Balkema, Rotterdam.

Morikawa, R., 1956, Fusulinids from Onagata, Kamiyoshida-mura, northern part of Kanto Mountainland. Science Reports of the Saitama University, Series B, 2, 249–260.

Morikawa, R. and Isomi, H., 1960, A new genus *Biwaella*, Schwagerina-like *Schubertella*. Science Reports of the Saitama University, Series B, 3, 301–305.

Morikawa, R. and Isomi, H., 1961, Studies of Permian fusulinids in the east of Lake Biwa, central Japan. Geological Survey of Japan, Report 191, 1–30.

Morikawa, R. and Kobayashi, N., 1960, Two new species of Okeatella from Kanto Massif, Japan. Science Reports of the Saitama University, Series B, 3, 307–312 with plate 55.

Nagai, H., Zhu, S. Z., Kametaka, M. & Wu C. Y. 1998. Preliminary Report on Middle Permian Radiolarians from the Gufeng Formation at Anmenkou, Chaohu City, Anhui province Province, China. Bulletin of the Nagoya University Furukawa Museum, 14, 115–123.

Nie, Z.T. and Song, Z.M., 1983a, Fusulinids of lower Permian Tunlonggongba Formation from Rutog of Xizang (Tibet province), China. Earth Science – Journal of Wuhan College of Geology, 1983(1), 43–55. (in Chinese with English abstract)

Nie, Z.T. and Song, Z.M., 1983b, Fusulinids of Lower Permian Maokouian Longge Formation from Rutog, Xizang (Tibet province), China. Earth Science – Journal of Wuhan College of Geology, 1983(1), 57–68. (in Chinese with English abstract)

Niko, S., Yamakita, S., Otoh, S., Yanai, S. & Hamada, T. 1987. Permian radiolarians from the Mizuyagadani Formation in Fukuji area, Hida Marginal Belt and their significance. Journal of the Geological Society of Japan, 93, 431–433. [In Japanese.]

Niu, Z.J. and Wu, J., 2016, Fusulinid Fauna of Permian Volcanic – Depositional Succession (Setting) in Southern Qinghai province, Norwest China. 199 pp. China University of Geoscience Publishing House, Wuhan (in Chinese with English summary)

Niwa, M., Hotta, K. and Tsukada, K., 2004. Middle Permian fusulinoideans from the Moribu Formation in the Hida-gaien Tectonic Zone, Nyukawa Village, Gifu Prefecture, central Japan. Journal of the Geological Society of Japan, 110, 384–387. (in Japanese with English abstract)

Ozawa, T. and Kobayashi, F., 1990. Carboniferous to Permian Akiyoshi Limestone Group. p. E1–E31 with plates 1 to 13. In: Organizing Committee Benthos ’90 (ed.) Fossil and Recent Benthic Foraminifera in Some Selected Regions of Japan. Guidebook for Field Trips Organized on the Occasion of Fourth International Symposium on Benthic Foraminifera, Sendai, 1990. Tohoku University, Sendai.

Pitakpaivan, K., 1966, Fusulines of the Rat Buri Limestone of Thailand. p. 63–155. In Kobayashi, T. and Toriyama, R. (eds.) Geology and Palaeontology of Southeast Asia. Volume 20. University of Tokyo Press, Tokyo.

Regional Geological Survey Team of Anhui province (ed.), 1982, Paleontological Atlas of Anhui province. Fusulinid Fossil from Anhui province. 145 pp. Anhui province Science and Technology Press, Anhui province. (in Chinese, title translated).

Rudenko, V. S. & Panasenko, E. S. 1997. Biostratigraphy of Permian deposits of Sikhote-Alin based on radiolarians. Pp. 73–84 in A. Baud, I. Popova, J. M. Dickins, S. Lucas, Y. Zakharov (eds) Late Paleozoic and Early Mesozoic Circum-Paciric Events: Biostratigraphy, Tectonic and Ore Deposits of Primoryie (Far East Russia). IGCP Project 272. Mémoires de Géologie (Lausanne), 30.

Sada, K. and Yokoyama, T., 1966, Upper Permian fusulinids from the Taishaku Limestone in west Japan. Transactions and Proceedings of the Palaeontological Society of Japan, New Series, 63, 303–315.

Saesaengseerung, D., Agematsu, S., Sashida, K. & Sardsud, A. 2009. Discovery of Lower Permian radiolarian and conodont faunas from the bedded chert of the Chanthaburi area along the Sra Kaeo sutue zone, eastern Thailand. Paleontological Research, 13, 119–138.

Sakagami, S. and Iwai, J., 1974, Fusulinacean fossils from Thailand, Part VIII. Permian fusulinaceans from the Pha Duk Chik Limestone and in the limestone conglomerate in its environs, north Thailand. p. 49–81. In Kobayashi, T. and Toriyama, R. (eds.) Geology and Palaeontology of Southeast Asia. Volume 14. University of Tokyo Press, Tokyo.

Sashida, K., 1995. Late Carboniferous and Early Permian radiolarian biostratigraphy in the chert block embedded in the Jurassic Kawai Formation, Kanto Mountains, central Japan. Ann. Rep., Inst. Geosci., Univ. Tsukuba. 21, 33–40.

Sashida, K., Igo, H., Hisada, K.I., Nakornsri, N., Ampornmaha, A., 1993. Occurrence of Paleozoic and Early Mesozoic Radiolaria in Thailand (preliminary report). Journal of Southeast Asian Earth Sciences 8, 97–108.

Sheng, J.Z., 1956, Permian fusulinids from Liangshan, Hanchung, southern Shensi. Acta Palaeontologica Sinica, 4, 175–227. (in Chinese with English abridged parts)

Sheng, J.Z., 1958, Some fusulinids from the Maokou Limestone of Chinghai Province, northwestern China. Acta Palaeontologica Sinica, 6, 268–291. (in Chinese with English abridged parts)

Sheng, J.Z., 1963, Permian fusulinids of Kwangsi, Kueichow and Szechuan. Palaeontologica Sinica, 149(New Series 10), 1–247. (in Chinese with English abridged parts)

Sheng, J.Z., 1965, Fusulinids from the western part of Hainan province Island, Kwangtung Province. Acta Palaeontologica Sinica, 13, 563–583. (in Chinese with English abridged parts)

Sheng, J.Z. and Sun, D.D., 1975, Fusulinids of Qinghai province. Geological Publishing House, Beijing, 92 pp. (in Chinese, title translated).

Sheng, J. Z. & Wang, Y. J. 1985. Fossil Radiolaria from Kufeng Formation at Longtan, Nanjing. Acta Palaeontologica Sinica, 24, 171–180.

Shi, Y.K., Yang, X.N. and Jin, X.C., 2005, Restudy of the “*Rugososchwagerina*” of the middle Permian from Xiaoxinzhai of Gengma, western Yunnan province. Acta Palaeontologica Sinica, 44, 535 – 544. (in Chinese with English abridged parts)

Shi, Y.K., Jin X.C., Huang, H. and Yang, X.N., 2008, Permian fusulinids from the Tengchong Block, western Yunnan province, China. Journal of Paleontology, 82, 118–127.

Shi, L., Feng, Q. L., Shen, J., Ito, T. & Chen, Z. Q. 2016. Proliferation of shallow-water radiolarians coinciding with enhanced oceanic productivity in reducing conditions during the Middle Permian, South China: evidence from the Gufeng Formation of western Hubei province Province. Palaeogeography, Palaeoclimatology, Palaeoecology, 444, 1–14.

Shi, Y.K., Huang, H. and Jin, X. C., 2017, Depauperate fusulinid faunas of the Tengchong Block in western Yunnan province, China, and their paleogeographic and paleoenvironmental indications. Journal of Paleontology, 91, 12–24.

Shimakawa, M. & Yao, A. 2006. Lower–Middle Permian radiolarian biostratigraphy in the Qinzhou area, South China. Journal of Geosciences, Osaka City University, 49, 31–47.

Sun, Q.L., 1993, Early Permian fusulinids from Huangyangling-Muztag Peak of Kunlun Mountains, Xinjiang province. Acta Micropalaeontologica Sinica, 10, 257–274. (in Chinese with English abstract).

Sun, Q.L. and Zhang, L.X., 1985, Early Permian fusulinids from Alge Mountain of Xinjiang province. Acta Palaeontologica Sinica, 24, 503–510. (in Chinese with English abstract)

Sun, Q.L. and Zhang, L.X., 1988, Early Permian fusulinids from the Kongkashan Pass of Xinjiang province. Acta Micropalaeontologica Sinica 5, 367–378 (in Chinese with English abstract)

Suyari, K., 1962, Geological and paleontological studies in central and eastern Shikoku, Japan. Part II. Paleontology. Journal of Gakugei, Tokushima UniversitJournal of Gakugei, Tokushima University, 12, 1–64.

Suyari, K., Kuwano, Y., Ishida, K., 1982. Stratigraphy and geological structure of the Mikabu Greenrock Terrain and its environs-II. Some informations about the Mesozoic stratigraphy of the North Subbelt of the Chichibu Belt. Journal of Science, University of Tokushima XV, 51–71. (in Japanese with English abstract)

Takaoka, Y., 1966, Fusulinid from the Mt. Tatoro, Mt. Kano, Mt. Futago and Mt. Shiraishi areas of the Kanto-massif, central Japan. Chichibu Museum of Natural History, 13, 39–70. (In Japanese with English description)

Tazawa, J. and Hasegawa, Y., 2007. Permian fusulinoideans from the Atagoyama Formation of Choshi, Chiba Prefecture, central Japan, and their tectonic significance. Journal of the Geological Society of Japan, 113, 406–416. (in Japanese with English abstract)

Toriyama, R., 1947, On some fusulinids from Tosayama, Koti-ken, Shikoku, with a note on the stratigraphic range of Neoschwagerina. Japanese Journal of Geology and Geography, 20, 63–82.

Toriyama, R., 1958, Geology of Akiyoshi. Part III. Fusulinids of Akiyoshi. Memoirs of the Faculty of Science, Kyushu University, Series D, Geology, 7, 1–264.

Toriyama, R., 1976, Fusuline fossils from Thailand, Part IX. Permian fusulines from the Rat Buri Limestone in the Khao Phlong Phrab area, Sara Buri, central Thailand. p. 1–116. In Kobayashi, T. and Hashimoto, W. (eds.) Geology and Palaeontology of Southeast Asia. Volume 17. University of Tokyo Press, Tokyo.

Toriyama, R. and Kanmera, K., 1977, Fusuline fossils from Thailand, Part X. The Permian fusulines from the Limestone Conglomerate Formation in the Khao Phlong Phrab area, Sara Buri, central Thailand. p. 1–27. In Kobayashi, T., Toriyama, R. and Hashimoto, W. (eds.) Geology and Palaeontology of Southeast Asia. Volume 18. University of Tokyo Press, Tokyo.

Toriyama, R. and Kanmera, K., 1979, Fusuline fossils from Thailand XII. Permian fusulines from the Ratburi Limestone in the Khao Khao area, Sara Buri, central Thailand. p. 23–93. In Kobayashi, T. and Toriyama, R. (eds.) Geology and Palaeontology of Southeast Asia. Volume 14. University of Tokyo Press, Tokyo.

Udchachon, M., Thassanapak, H. & Burrett, C. 2018. Early Permian radiolarians from the extension of the Sa Kaeo Suture in Cambodia–tectonic implications. Geological Magazine, 155, 1449–1464.

Ueno, K., 1991, Early evolution of the families Verbeekinidae and Neoschwagerinidae (Permian Fusulinacea) in the Akiyoshi Limestone Group, Southwest Japan. Transactions and Proceedings of the Palaeontological Society of Japan, New Series, 164, 973–1002.

Ueno, K., 1992. Verbeekinid and neoschwagerinid fusulinacean from the Akiyoshi Limestone Group above the Parafusulina kaerimizensis Zone, Southwest Japan. Transactions and Proceedings of the Palaeontological Society of Japan, New Series, 165, 1040–1069.

Ueno, K., 2003. The Permian fusulinoidean faunas of the Sibumasu and Baoshan blocks: their implication for the paleogeographic and paleoclimatologic reconstruction of the Cimerian continent. Palaeogeography, Palaeoclimatology, Palaeoecology, 193, 1–24.

Ueno, K. and Sakagami, S., 1993, Middle Permian foraminifers from Ban Nam Suai Tha Sa-At, Changwat Loei, northeast Thailand. Transactions and Proceedings of the Palaeontological Society of Japan, New Series, 172, 277–291.

Ueno, K. and Tazawa, J., 2004. Monodiexodina from the Permian Oguradani Formation, Hida gaien Belt, central Japan. Science Reports, the Niigata University, 19, 25–33.

Ueno, K., Tazawa, J. and Miyake, Y., 2006, Middle Permian fusulinoideans from Hatahoko in the Nyukawa area, Gifu Prefecture, Mino Belt, central Japan. Science Reports, the Niigata University (Geology), 21, 47–72.

Ujiié, H., Oba, T., 1991. Geology and Permo-Jurassic Radiolaria of the Iheya Zone, Innermost Belt of the Okinawa Islands region, middle Ryukyu island arc, Japan. Part 1: Geology and Permian Radiolaria. Bulletin of College Science, University of Ryukyus 51, 35–55.

Wang, R. J. 1993. Fossil Radiolaria from Kufeng Formation of Chaohu, Anhui province. Acta Palaeontologica Sinica, 32, 442–457. [In Chinese with English abstract.]

Wang, K.L. and Sun, X.F., 1973, Carboniferous and Permian foraminifera of the Chinling Range and its geologic significance. Acta Geologica Acta, 1973(2), 138–163, 171–178. (in Chinese with English abridged parts)

Wang, J.H. and Tang, Y., 1986. Fusulinids from Chihsia Formation of Lengwu district in Tonglu County, Zhejiang province. Acta Micropalaeontologica Sinica, 3, 3–12. (in Chinese with English abstract)

Wang, Y.J. and Zhou, J.P., 1986, New material of fusulinids from Xainza, Xizang. Bulletin of the Nanjing Institute of Geology and Paleontology, Academy of Sinica, 10, 142–156. (in Chinese with English abridged parts)

Wang, Y.J., Sheng, J.Z., Zhang, L.X., 1982a, Fusulinids from Xizang of China. In Scientific Expedition Team to the Qinghai province-Xizang Plateau, Chinese Academy of Science (ed.) Paleontology of Xizang. Book 3. Scientific Publishing House, Beijing, pp. 1–80. (in Chinese with English abridged parts)

Wang, Y.S., Wang, L.L., Wang, J.H., Zhu, Z.G., Ling, G.W., Zhang, L.X. and Qiang, Q., 1982b, Fusulinida. p. 5–108, 423–495 with plates 1 to 30. In Nanjing Institute of Geology and Mineral Resources (ed.) Paleontological Atlas of East China (2). Volume of Late Paleozoic. Geological Publishing House, Beijing. (in Chinese)

Wu, H. R., Xian, X. Y. & Kuang, G. D. 1994. Late Paleozoic radiolarian assemblages of southern Guangxi province and its geological significance. Scientia Geologica Sinica, 29, 339–345. [In Chinese with English abstract.]

Xia, G.Y., 1994, The study of Carboniferous-Permian fusulinid fossils from Longyin, Puan, Guizhou province. Professional Papers of Stratigraphy and Paleontology, 25, 141–167. (in Chinese with English abstract)

Xie, S.G., 1982. Protozoa. In Geological Bureau of Hunan province (ed.) The Palaeontological Atlas of Hunan province. Geological Memoirs, Series 2 and Number 1. Geological Publishing House, Beijing, pp. 2–73. (in Chinese).

Yao, A., Kuwahara, K., Ezaki, Y., Liu, J. B. & Hao, W. C. 2004. Permian radiolarians from the Qinfang Terrane, South China, and its geological significance. Journal of Geosciences, Osaka City University, 47, 71–83.

Yang, Z.D., 1985. Restudy of fusulinids from the “Maokou Limestone” (Permian) at Datiguan, Langdai Guizhou province. Acta Micropalaeontologica Sinica, 2, 307–338. (In Chinese with English abstract).

Yu, J., 1996. Permian radiolarian biostratigraphy in the Guizhou province Area, China. Journal of Geosciences, Osaka City University 39, 123–135.

Zhang, L.X., 1982, Fusulinids of eastern Qinghai province–Xizang Plateau. In Regional Geological Survey Team of Geological Bureau of Sichuan province and Institute of Nanjing Geology and Paleontology, Academy of Science (eds.) Stratigraphy and Palaeontology in Western Sichuan province and Eastern Xizang, China. Part 2. Sichuan province People Press, Chengdu, 322pp. (in Chinese with English abstract).

Zhang, Z.H. and Hong, Z.Y., 2000, Discovery of the *Staffella* fauna from Chihsia Formation’s bottom in southeastern Hunan province. Journal of Quanzhou Normal College (Natural Science), 18, 21–32.

Zhang, L.X. and Wang, Y.J., 1974, Permian Fusulinida. In Nanking Instutute of Geology and Paleontology, Academia Sinica (ed.) Handbook of the Stratigraphy and Paleontology in Southwest China. Science Press, Beijing, pp. 289–296. (in Chinese).

Zhang, Y.C., Wang, Y., Shen, S.Z., 2009, Middle Permian (Guadalupian) fusulines from the Xilanta Formation in the Gyanyima area of Burang County, southwestern Tibet province, China. Micropaleontology, 55, 463–486.

Zhang, L.X., Zhou J.P. and Sheng, J.Z., 2010, Upper Carboniferous and lower Permian fusulinids from western Guizhou province. Palaeontologia Sinica, New Series, 195(34), 1–244. (in Chinese with English description).

Zhang, Y.C., Wang, Y., Zhang, Y.J. and Yuan, D.X., 2013, Artinskian (Early Permian) fusuline fauna from the Rongma area in northern Tibet province: palaeoclimatic and palaeobiogeographic implications. Alcheringa, 37, 529–546.

Zhang, Y.C., Shi, G.R., Shen, S.Z. and Yuan, D.X., 2014. Permian fusuline fauna from the lower part of the Lugo Formation in the central Qiangtang Block and its geological implications. Acta Geologica Sinica (English Edition), 88, 365–379.

Zhang, Y.C., Shen, S.Z., Zhang, Y.J., Zhu, T.X., An, X.Y., Huang, B.X., Ye, C.L., Qiao F. and Xu, H.P., 2019. Middle Permian foraminifers from the Zhabuye and Xiadong areas in the central Lhasa Block and their paleobiogeographic implications. Journal of Asian Earth Sciences, 175, 109–120.

Zhang, Y.C., Aung, K.P., Shen, S.Z., Zhang, H., Zaw, T., Ding, L., Cai, F.L., Sein, K., 2020. Middle Permian fusulines from the Thitsipin Formation of Shan State, Myanmar and their palaeobiogeographical and palaeogeographical implications. Papers in Palaeontology, 6, 293–327.

Zhou, T.M., 2001, Middle Permian strata and fusulinid zoning in Puer area. Yunnan province Geology, 20, 297–307. (in Chinese)

Zhou, J.P. and Zhang, L.X., 1984, Fusulinids from the Chihsia Formation of Mt. Qixiashan, Nanjing. Acta Palaeontologica Sinica, 23, 716–724. (in Chinese with English abstract)

Zhou, T.M., Sheng, J.Z. and Wang, Y.J., 1987, Carboniferous–Permian boundary beds and fusulinid zones at Xiaodushan, Guangnan, eastern Yunnan province. Acta Micropalaeontologica Sinica, 4, 123–160. (in Chinese with English abridged parts)

**Supplement 4. The morphological terminology for the albaillellids in Figure 3, Supplement 5 and 6.**


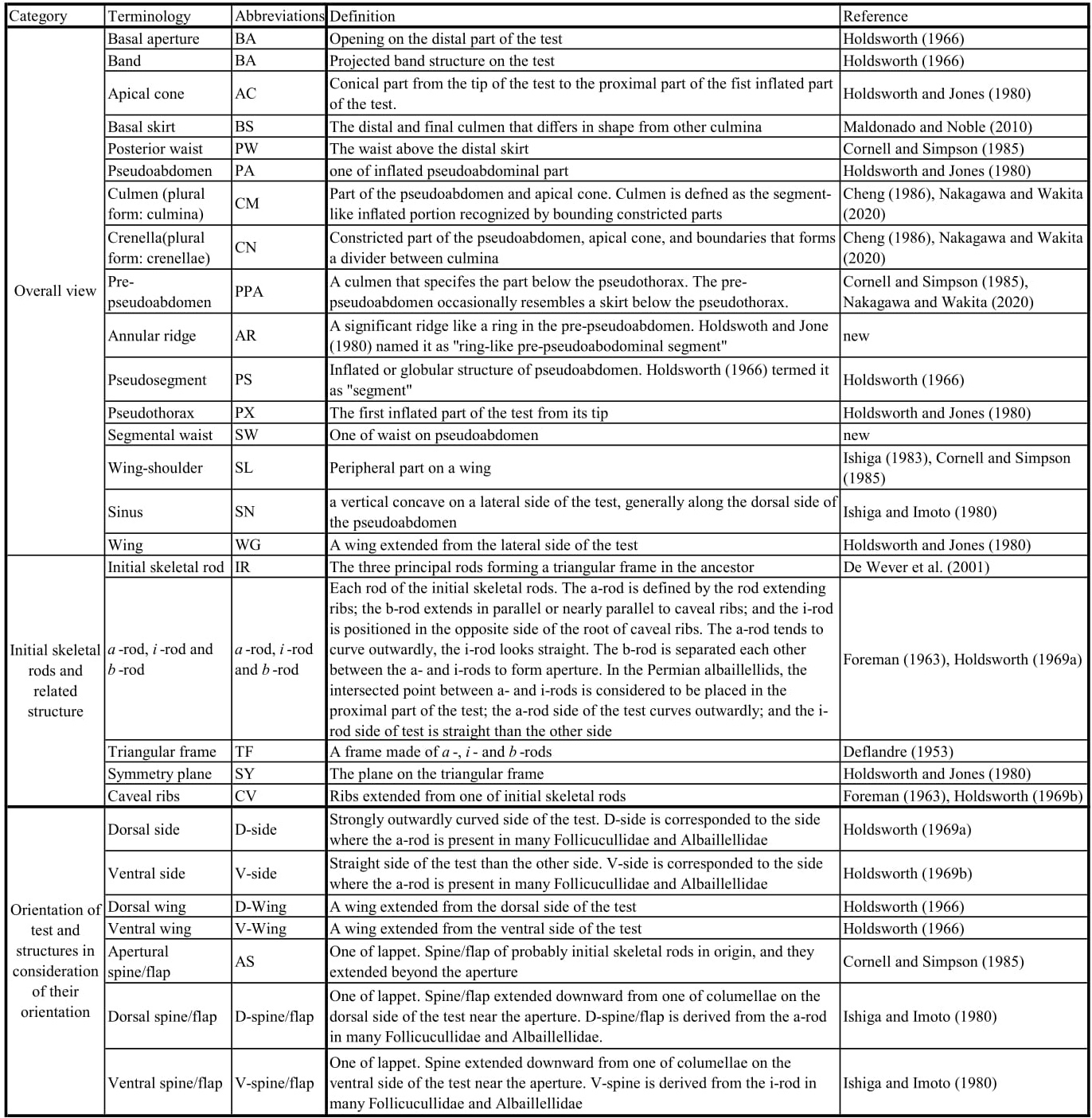


**Supplement 5. Systematic Palaeontology.**

The general morphological terminology for Permian albaillellids is summarized in supplement 4. Undulation patterns in the main species of *Longtanella* are shown in Figure 3. The specimens described herein are registered and deposited in the China University of Geosciences, Wuhan. We also include an updating of the genus concept since the paper of Xiao *et al.* (2020) was published.

Order **Albaillellaria** Deflandre, 1953, emend. Holdsworth, 1969

Family **Follicucullidae** Ormiston & Babcock, 1979

Follicucullidae. Ormiston & Babcock, 1979, p. 332; Holdsworth & Jones, 1980, p. 285; Kozur, 1981, p. 265; Nazarov, 1988, p. 83; Kozur & Mostler, 1989, p. 169-170; Cordey, 1998, p. 37; Afanasieva, 2000, p. 124-125; De Wever *et al.*, 2001, p. 93, 95; Afanasieva, 2002, p. 28; Afanasieva *et al.*, 2005, p. S270; Afanasieva & Amon, 2006, p. 105.

Holdsworthellidae. Kozur, 1981, p. 268.

Follicullidae [sic] (=Follicucullidae). Petrushevskaya, 1984, p. 125.

Pseudoalbaillellidae. (nomen nudum) Cheng, 1986, p. 45; (nomen nudum) Wang & Qi, 1995, p. 380.

Follicucullinae. Kozur & Mostler, 1989, p. 170-171 [in Follicucullidae]; Afanasieva, 2000, p. 125 [in Follicucullidae].

Holdsworthellinae. Kozur & Mostler, 1989, p. 182-183 [in Follicucullidae].

Remarks. The Paleozoic Genera Working Group organized by the International Association of Radiolarists (InterRad) decided to include only three genera, *Follicucullus*, *Ishigaconus* and *Parafollicucullus*, in the Follicucullidae (Caridroit *et al.* 2017; Noble *et al.* 2017). Soon after this decision, several claims arose from several non-member specialists about this excessive synonymization (Ito 2020; Nakagawa & Wakita 2020; Nestell & Nestsell 2020). Quantitative and parsimony methods as an objective test of the genus-level classification resulted in a scheme of ten genera (*Cariver*, *Curvalbaillella*, *Follicucullus*, *Haplodiacanthus*, *Holdsworthella*, *Kitoconus*, *Longtanella*, *Parafollicucullus* *sensu stricto*, *Parafollicucullus* in Lineage I (= *Parafollicuculinoides* gen. nov. in this paper), and *Pseudoalbaillella* *sensu stricto*) (Xiao *et al.* 2020, Fig. 5).

Genus ***Parafollicucullinoides*** Xiao, Ito & Suzuki, gen. nov.

Type species. *Pseudoalbaillella globosa* Ishiga & Imoto in Ishiga *et al.* (1982).

Included species (written as an original described name). *Pseudoalbaillella lomentaria* Ishiga & Imoto, 1980; *Pseudoalbaillella ornata* Ishiga & Imoto, 1980; *Pseudoalbaillella longtanensis* Sheng & Wang, 1985; *Pseudoalbaillella yanaharensis* Nishimura & Ishiga, 1987.

Diagnosis. Shell divided into apical cone, pseudothorax, pre-pseudoabdomen and segmented pseudoabdomen (culmina and crenellae). Apical cone straight or slightly curved, about 1/3 of the length of the test. Pseudothorax globular and usually inflated. Two blade-like wings long and wide. Pre-pseudoabdomen waist short. Pseudoabdomen with two or more culmina similar in height.

Derivation of name. Named for its similarity to *Parafollicucullus*, but different.

Range and distributions. Permian, Late Sakmarian to middle Capitanian.

Remarks. The new genus contains the species of ‘*Parafollicucullus*’ in lineage I in Xiao *et al.* (2020). They differ from the true *Parafollicucullus* of lineage Ⅱ in having a long straight apical cone, inflated pseudothorax, and segmented pseudoabdomen.

Genus ***Curvalbaillella*** Kozur & Mostler, 1989, sensu emend. Xiao & Suzuki herein

*Curvalbaillella*. Kozur & Mostler, 1989, p. 179–180; Noble *et al.*, 2017, p. 427–428.

*Kitoconus*. Kozur & Mostler, 1989, p. 178; Catalano *et al.*, 1989, p. 95.

Type species. *Pseudoalbaillella u-forma* Holdsworth & Jones, 1980.

Revised diagnosis. Shell divided into apical cone, pseudothorax, and unsegmented pseudoabdomen. Apical cone short, straight or slightly curved. Pseudothorax globular and not inflated, less than 1/5 of the length of the test. Two hemicyclic-like wings short to medium in length which extend horizontally with straight shoulders. Pseudoabdomen unsegmented, almost invariant in width, straight or curved, sometimes the basal part of the test forms ‘U’-shape. Two flaps of the same size at the aperture, often extending in different directions (horizontal or upwards).

Remarks. Statistical test made by Xiao *et al.* (2020) pointed to the conspecific relationship of *Curvalbaillella* and *Kitoconus*. The difference between these genera is in the curved (*Curvalbaillella*) or straight (*Kitoconus*) pseudoabdomen. The known *Curvalbaillella* species (*Pseudoalbaillella bulbosa* Ishiga, 1982; *Pseudoalbaillella chilensis* Ling & Forsythe, 1987; *Pseudoalbaillella u-forma* Holdsworth & Jones, 1980; and *Pseudoalbaillella u-forma reflexa* Ling & Forsythe, 1987, in the original description) do not look like intermediate species for the *Kitoconus* genus (*Pseudoalbaillella elegans* Ishiga & Imoto, 1980 and *Pseudoalbaillella elongata* Ishiga & Imoto, 1980, in the original description) as was discussed by Kozur and Mostler (1989) . Some species identified as ‘*Pseudoalbaillella elegans*’ show a somewhat curved pseudoabdomen (eg., pl. 11, figs 10, 13, pl. 13, figs 11, 12 in Wang et al. 2012), supporting the statistically objective opinion of Xiao *et al.* (2020). Considering that the only obvious difference of *Kitoconus* and *Curvalbaillella* is the curvature of pseudoabdomen, we regard them as synonyms. Both *Kitoconus* and *Curvalbaillella* were established in the same paper (Kozur & Mostler 1989), but *Curvalbaillella* has objective priority over *Kitoconus* because the latter was established as a subgenus of the genus *Pseudoalbaillella*.

Genus ***Pseudoalbaillella*** Holdsworth & Jones, 1980 sensu emend. Xiao & Suzuki herein

*Pseudoalbaillella*. Holdsworth & Jones, 1980, p. 285; Noble *et al.*, 2017, p. 430.

Type species. *Pseudoalbaillella scalprata* Holdsworth & Jones, 1980.

Included species (written as an original described name). *Pseudoalbaillella rhombothoracata* Ishiga & Imoto, 1980; *Pseudoalbaillella scalprata* m. *postscalprata* Ishiga 1983; *Pseudoalbaillella scalprata praescalprata* Kozur 1989; *Pseudoalbaillella triangularis* Wang *et al.*, 2012; *Pseudoalbaillella desmoinesiensis* Nestell *et al.*, 2012.

Revised diagnosis. Shell divided into apical cone, pseudothorax, and unsegmented pseudoabdomen. Apical cone strongly curved. Pseudothorax triangular or rhombohedral, usually inflated, wider than pseudoabdomen and more than 1/3 of height of the test. Two long and wide blade-like wings which are oriented downwards with arc-shaped shoulder. Pseudoabdomen unsegmented, almost invariant in width and similar to pseudothorax in height. Two flaps of the different size at the arc-shaped basal aperture, often extending downwards vertically or obliquely.

Remarks. Holdsworth and Jones (1980) established *Pseudoalbaillella* and *Parafollicucullus*, and in the origin descriptions, *Parafollicucullus* differs from *Pseudoalbaillella* only for possessing a ring-like pre-pseudoabdominal segment between pseudothorax and pseudoabdomen. Later Kozur (1981) proposed *Pseudoalbaillella* is a synonym of *Parafollicucullus* because he thought these radiolarians usually having ring-like pre-pseudoabdominal segment. Most researchers agree with that these two genera are synonyms (Ishiga *et al.* 1982; Sheng & Wang 1985; De Wever *et al.* 2001; Nestell *et al.* 2012; Noble *et al.* 2017), except for which name has the priority. However, Statistical test made by Xiao et al. (2020) implied that *Pseudoalbaillella* could be distinguished from *Parafollicucullus* in both morphology (eg., the size and shape of apical cone and pseudothorax of *Pseudoalbaillella* are much greater than *Parafollicucullus*) and phylogeny (they belong to different lineages). Their taxonomic and phylogenetic analysis supports that *Follicucullus* is more related to *Pseudoalbaillella*, compared to *Parafollicucullus.* Thus, the definition of *Pseudoalbaillella* is revised herein, and separated from *Parafollicucullus.*

Genus ***Parafollicucullus*** Holdsworth & Jones, 1980 sensu emend. Xiao & Suzuki herein

*Parafollicucullus*. Holdsworth & Jones, 1980, p. 285; Noble *et al.*, 2017, p. 430.

Type species. *Parafollicucullus fusiformis* Holdsworth & Jones, 1980.

Revised diagnosis. Shell divided into apical cone, pseudothorax, and segmented pseudoabdomen. Apical cone slightly curved. Pseudothorax globular, less than 1/5 of the height of the test. Two symmetric narrow blade-like wings which are oriented downwards with straight shoulder. Pseudoabdomen with variable number of segments or bands, widest in the middle part. Pre-pseudoabdomen between the pseudothorax and pseudoabdomen, often constricted. Two flaps at the arc-shaped basal aperture.

Remarks. The concept of *Parafollicucullus* are expanded here, keeping its original important character “pre-pseudoabdominal segment”. The species of *Parafollicucullus* include the species of ‘*Parafollicucullus*’ in lineage II in Xiao *et al.* (2020).

Genus ***Follicucullus*** Ormiston & Babcock, 1979

*Follicucullus*. Ormiston & Babcock, 1979, p. 332; Noble *et al.*, 2007, p. 428.

*Ishigaconus*. Kozur & Mostler, 1989, p. 181; Noble *et al.*, 2007, p. 429.

Type species. *Follicucullus ventricosus* Ormiston & Babcock, 1979.

Remarks. Noble *et al.* (2017) favoured the opinion of Kozur & Mostler (1989) who separated *Ishigaconus* (type species: *Follicucullus scholasticus* Ormiston & Babcock, 1979) from *Follicucullus* (type species: *Follicucullus ventricosus*) because of a lack of any inflation of the pseudothorax in *Follicucullus scholasticus*. The statistical results in Xiao *et al.* (2020, figs. 3, 4) showed a difference between *F. ventricosus* and *F. scholasticus*, confirming the opinion of Kozur & Mostler (1989). However, the difference scores are small in Xiao *et al.* (2020, fig. 3), and if we accept this score as a clustering line, the Follicucullidae should be subdivided into 12 genera under the HQT-II statistical method, but this distinction cannot be supported by time-calibrated phylogenetic trees shown by Xiao *et al.* (2020, fig. 4). In consideration of the objective taxonomic scheme in Xiao *et al.* (2020), we apply the original concept of *Follicucullus*, with *Ishigaconus* as junior synonym.

Genus ***Longtanella*** Sheng & Wang, 1985 sensu emend. Ito, 2020, sensu emend. Xiao, Ito & Suzuki herein

*Longtanella* Sheng & Wang, 1985, p. 175; Kozur & Mostler, 1989, p. 179; Ito *et al.*, 2019, p. 270; Ito, 2020, p. 1–10.

Type species. *Longtanella zhengpanshanensis* Sheng & Wang, 1985

Revised diagnosis. Shell smooth, straight, bilaterally symmetrical or slightly asymmetric. The shell divided into apical cone, pseudothorax, pre-pseudoabdomen and pseudoabdomen. Apical cone and pseudothorax small, wings retrograde to bulge-shaped or absent. Pre-pseudoabdomen long and slender. Pseudoabdomen with or without undulation. In some specimens, four flaps present. These four flaps can be divided into two groups: a pair of large ones at front and back sides, and a pair of small ones at left and right sides (see Fig. 3I), extending vertically or a little obliquely from apertural margin downward.

Occurrences. The upper part of the Gufeng Formation in Chaohu City, Anhui province (Wang 1993; Nagai *et al.* 1998; Kametaka *et al.* 2009; Ito *et al.* 2013), Qinzhou city, Guangxi province province (Xia & Zhang 1998; Wang *et al.* 2012; this study), Hubei province province (Feng 1992) and Longtan region in Nanjing, South China (Sheng & Wang 1985); ophiolite complex in Qinghai province, western China (Li & Bian 1993); bedded chert of the Sra Kaeo Suture Zone in eastern Thailand (Saesaengseerung *et al.* 2009); Bentong-Raub Suture Zone in Malaysia (Metcalfe *et al.* 1999; Spiller 2002); Japan (Niko *et al.* 1987; Kashiwagi & Isaji 2015); Far East of Russia (Rudenko & Panasenko 1997).

Ranges. Kungurian to Wordian.

Remarks. The original definition of *Longtanella* is ‘*Shell smooth, straight, bilaterally symmetrical turriformis. The shell wall divided into the spire, the turri-body, the turri-body and the turri-bottom composed of ring-like swollen segments, last segment constrictive with 4 flaps vertically extending downward*’ (Sheng & Wang 1985, p. 179) and its diagnosis was revised as ‘*Follicucullidae having a smooth, straight, bilaterally symmetrical, and turriform shell composed of a conical proximal spired part and a bladder, with or without a skirt*’ (Ito 2020). Because our specimens also have similar overall appearances with *Longtanella*, we redefined *Longtanella* and included our specimens in this genus. Ito (2020) applied different morphological terminology such as ‘spired part’, ‘bladder’ and ‘skirt’. Our paper, though Ito is a co-author, prioritized the more common general terms that are applicable to any follicucullids (see supplement 6 for the general characters of Follicucullidae).

The fundamental difference of *Longtanella* from all other Follicucullidae genera is in its symmetrical morphology on the front-back view and the ‘dorsal-lateral view’. This character excludes confusion with poorly-preserved specimens of any other genera. Noble *et al.* (2017, p. 430) synonymized *Longtanella* with ‘*Parafollicucullus*’ by ambiguous reasoning, namely, ‘*this genus fits within the parameters of Parafollicucullus and should be treated as a junior synonym*.’ Ito (2020) proved the independence of *Longtanella* by observation of topotypes, and Xiao *et al.* (2020) confirmed this validity by statistical methods. Besides our standpoints and those of the original authors (Sheng & Wang 1985), the validity of *Longtanella* is accepted in many reports on Asian Permian radiolarians (Rudenko 1991; Nagai *et al.* 1998; Xia & Zhang 1998; Kametaka *et al.* 2009; Saesaengseerung *et al.* 2009; Muhanmad Ashahadi *et al.* 2016). By contrast, studies in North America are likely to reject its validity (Cordey 1998; Nestell & Nestell 2010; Nestell *et al.* 2012; Noble *et al.* 2017). This contradiction may be because North America, except for British Columbia, lies outside the *Longtanella* territory.

The orientation of the follicucullid test is defined by Cartesian coordinates with the ventral-dorsal side, front-back side and top-bottom side axes (Nakagawa & Wakita 2020). This scheme needs to be slightly revised because *Longtanella* shows a symmetrical appearance with respect to the dorsal-ventral and the front-back axes. Taking into account of the realistic recognition of shell orientation, we simply apply the terms left-right side instead of dorsal-ventral side. Front-back side (Fig. 3A) as well as top-bottom side are simply applied for the external display.

The genus *Longtanella* was originally included in the family Albaillellidae by Sheng & Wang (1985). The Albaillellidae has visible caveal ribs from the *a*-rod side (De Wever *et al.* 2001, p. 92) but *Longtanella* lacks these ribs. Feng (1992) depicted two *Longtanella* species, *Longtanella mengshengensis* and *Longtanella turgida* in the *Parafollicucullus fusiformis* assemblage, but he only described *L. turgida* but not *L. mengshengensis*. Subsequently *L. mengshengensis* is a *nomen nudum*.

***Longtanella lanceoliformis*** Xiao & Suzuki sp. nov.

(Figs 3E, S2A–P)

1991 *Follicucullus scholasticus* Ormiston & Babcock; Ujiie & Oba: pl. 3, fig. 2.

1993 *Pseudoalbaillella* sp. H; Li & Bian: 416, 417, pl. 1, fig. 11.

1994 *Pseudoalbaillella* sp. B; Wu *et al.*: pl. 2, fig. 7.

Diagnosis. *Longtanella* with ‘short spear’ lance-like shell.

Derivation of name. Latin adjective, *lanceola* + *formis* (-is, -e), meaning lance-formed. Noun.

Material. Holotype, Fig. S2M, specimen ST 1-2_i026; paratype, Fig. S2D, specimen ST1-2_i001. 21 specimens were examined by SEM and 16 of them are illustrated.

Measurements (μm). Height of shell 223–357 (mean 279); height of AC 12–35 (mean 21); width of the basal AC 17–39 (mean 29); height of PPA 34–76 (mean 61); height of PX 26–63 (mean 42); width of PX 22–64 (mean 40); height of PA 130–214 (mean 162); based on 19 specimens.

Occurrences. Middle Kungurian–Roadian; Ophiolite complex and the Bancheng Formation of South China, the Southern Chichibu Belt of Japan (Ujiie & Oba 1991).

Description. Shell smooth, consisting of inflated oblong apical (AC) to pseudothoracic (PX) part, long thin pre-pseudoabdomen (PPA), and relatively wider pseudoabdomen (PA). AC very short (<1/2 of the PX height), straight, pyramidal with an apical spinulus. AC gradually joining with PX. PX slightly inflated without wings, forming ‘spearhead’ of a lance together with AC. No distinguishable pseudothoracic bulb in PX. The distal part of PX obviously narrowing to connect with PPA. PPA long and slender (1.3 to 1.5 times longer than the height of AC-PX part and 1/2 to 4/5 of the maximum width of PX). Pre-pseudoabdominal junction is easily distinguishable but does not form stricture. PA cylindrical and distally widening, straight. The crenelle between the distal end of PA and basal skirt (BS) is variable from smooth to necked connection. Some specimens with distinguishable BS is curved to the dorsal side. Four flaps vertically or little obliquely extending from apertural margin downward (Fig. S2M).

Remarks. This new species is similar to *Follicucullus porrectus* Rudenko in Belyansky *et al.*, 1984 in having a slender and smooth shell, but the new species is distinguished from the latter by the spearhead-like apical cone–pseudothoracic part and the presence of four flaps on the basal skirt. According to Nestell *et al.* (2019, p. 278), *Follicucullus japonicus* Ishiga, 1991 has a wider pseudothorax, a shorter and not cylindrical pseudoabdomen and two distinctive apertural flaps whereas *F. porrectus* lack apertural flaps. Besides the validity discussion on *F. japonicus*, no similar morphotypes with four flaps have been known in *Follicucullus*.

***Longtanella edamame*** Xiao & Suzuki sp. nov.

(Figs 3D, S2Q–X)

Diagnosis. *Longtanella* having edamame-shaped pseudoabdomen.

Derivation of name. Edamame, noun, meaning a kind of soybean in Japanese. It literally means ‘stem beans’ with a few undulating zones. This species is named for the edamame-like outline of the pseudoabdomen.

Material. Holotype, Fig. S2Q, specimen 13ST15-4_148; paratype, Fig. S2W, specimen 13ST6-1_245. 12 specimens were examined by SEM and eight of them are illustrated.

Measurements (μm). Height of shell 216–340 (mean 270); height of AC 15–49 (mean 34); width of the basal AC 18–36 (mean 24); height of PPA 22–87 (mean 43); height of PX 22–58 (mean 38); width of PX 26–55 (mean 43); height of PA 109–270 (mean 183); based on 12 specimens.

Occurrences. Middle Kungurian–Roadian; Bancheng Formation of South China.

Description. Shell consisting of an acute apical cone, small rhomboidal to simple inflated pseudothorax with wing-like acute edges in its both sides, short and thin pre-pseudothorax, two to three undulated segments on pseudothorax with basal skirt. PX is generally rhombic in outline but it also appears as a laterally facing oblong pseudothoracic bulb. PX is separated from PPA in the case of smaller PX with a stricture whereas from PPA in the case of rhombic PX with a distinguishable connection. PPA cylindrical with or without one annular ridge (Fig. S2W, X for annular ridge). Height of PPA is nearly similar throughout shell and its height is variable from 22 μm in the case of no annular ridge to 87 μm in the case of the presence of distinctive annular ridge. PA joined with PPA by a significant stepped shoulder. PA is fusiform in shape with three crenellae to form an edamame-like outline. BS cylindrical, usually curving to ventral side.

Remarks. This new species is unique in exhibiting morphological instability in the distinction between the pre-pseudothorax and upper part of the pseudothorax. Some specimens (e.g. Fig. S2U) look as if they have a short pre-pseudothorax with thinner upper pseudothorax or a stepped pre-pseudothorax. Others (e.g. Fig. S2W, X) look as if they have two crenellae and one culmen as a long and thin pre-pseudothorax. In consideration of these unstable morphological characters, the proximal end of the pseudothorax is determined by the proximal end of the ‘spindle’ form (Fig. S2W, X). This new species is similar to *Parafollicucullus longtanensis* (Sheng & Wang, 1985) in having a similar shell, but the latter has an undulated pseudothorax with two long wings.

***Longtanella* *jingyi*** Xiao & Suzuki sp. nov.

(Fig. S3D–G)

2016 *Longtanella*? sp. A; Ito *et al.*: fig. 4.5.

Diagnosis. *Longtanella* with a rhombic pseudothorax with tiny wings and elegant shaped smooth pseudoabdomen, and symmetric shell.

Derivation of name. Chinese word ‘jing’ and ‘yi’, meaning ‘excellent’ and ‘beautiful’, respectively, for a lady.

Material. Holotype, Fig. S3F, specimen ST1-3_i053; paratype, Fig. S3D, specimen ST1-3_061; Eight specimens were examined by SEM and four of them are illustrated.

Measurements (μm). Height of shell 123–250 (mean 217); height of AC 23–58 (mean 36); width of the basal AC 21–40 (mean 27); height of PPA 19–64 (mean 40); height of PX 24–52 (mean 32); width of PX 31–63 (mean 50); height of PA 95–150 (mean 123); based on eight specimens.

Occurrences. Middle Kungurian–Roadian; Bancheng Formation of South China and pebble of the Kamiaso Unit of the Mino-Tamba-Ashio Belt of Japan.

Description. Shell consisting of a tiny apical cone, rhombic pseudothorax, pre-pseudoabdomen and smooth pseudoabdomen without undulation. AC with acute apex, similar in the height with PX; PX a little inflated with very short wings, forming an arrow shape together with AC. PPA cylindrical, growing wider downwards with no constriction with the PA, usually 1.2 times longer than the height of PX. PA fusoid and smooth without undulation. Posterior waist reduced in both dorsal and ventral sides; basal skirt short with flaps.

**Remarks.** The taxonomic position of this new species at the genus level could be confused with a poorly preserved *Parafollicucullus* by loss of the wings if we refer to *Parafollicucullus corniculatus* (Rudenko & Panasenko, 1990) and *Parafollicucullus internata* (Wang in Wang *et al.*, 2012). However, *Longtanella jingyi* sp. nov. differs from any *Parafollicucullus* species by its symmetrical shell. This new species is probably homeomorphic with some *Parafollicucullus* (eg. *Pa. corniculatus* and *Pa. internata*). However, the small acute apical cone and rhombic pseudothorax without wings in *L. jingyi* sp. nov. form an arrow shape, and subsequently makes it easy to differentiate from the *Parafollicucullus* species with a distinct longer cone-shaped AC. This new species differs from *Longtanella* cf. *edamame* sp. nov. in having a smooth pseudoabdomen without undulation.

***Longtanella*** ***tokkuriformis*** Xiao & Suzuki sp. nov.

(Figs 3E, S3H–O)

2015 *Pseudoalbaillella* sp. cf. *P. monacantha*; Kashiwagi & Isaji: 42, figs 5.1–5.4.

2016 *Follicucullus monacanthus* Ishiga *et al.*; Shi *et al.*: fig. 4.16.

2016 *Follicucullus dilatatus* Belyansky; Shi *et al.*: fig. 4.17.

2020 *Longtanella* sp. B; Ito: figs 6F–6J.

Diagnosis. *Longtanella* with strongly constricted pre-pseudoabdomen and strongly inflated oblong pseudoabdomen.

Derivation of name. Noun. Tokkuri is a Japanese traditional small bottle with strong neck and large body for alcohol.

Material. Holotype, Fig. S3L, specimen ST1-3_i045; paratype, Fig. S3J, specimen 13ST1-4_072. 19 specimens were examined by SEM and 8 of them are illustrated.

Measurements (μm). Height of shell 136–247 (mean 200); height of AC 15–51 (mean 31); width of the basal AC 14–31 (mean 24); height of WA 19–55 (mean 33); height of PX 24–45 (mean 34); width of PX 29–58 (mean 41); height of PA 79–152 (mean 116); based on 17 specimens.

Occurrences. Middle Kungurian–Roadian; Gufeng and Bancheng Formation of South China and Permian pebbles in the Lower Cretaceous Choshi Group of the Kurosegawa Belt in Japan.

Description. Shell consisting of conical apical cone, small pseudothorax, strongly constricted pre-pseudoabdomen, and strongly inflated oblong and smooth pseudoabdomen without any undulation. AC is conical in shape and its shape varies from thin acute (Fig. S3H, K, L) to thick acute (Fig. S3M, N). PX slightly inflated with rudimentary wings. PPA is cylindrical and widens downwards. PA particularly wide, sharply increasing downwards, stocky in the bottom; no constriction existed between the pseudothorax and basal aperture. More than two flaps visible but the exact number is unknown.

Remarks. This new species has a wide shell body. This new species was illustrated as *Parafollicucullus monacanthus* (Ishiga and Imoto in Ishiga *et al.*, 1982) or *Follicucullus dilatatus* Rudenko in Belyanskiy *et al.*, 1984 in previous papers (Kashiwaki & Isaji, 2015; Shi *et al.* 2016). These two species are different from *Longtanella tokkuriformis* sp. nov., because *Pa. monacanthus* has one tiny wing on the dorsal side (e.g. Ito et al. 2015) and the pseudothorax of *F. dilatatus* is not obvious and inflated, compared to the new species. The new species is also similar to *Parafollicucullus bella* (Sheng & Wang, 1985) in the shell outline, but the latter has a distinguishable basal skirt with obvious constriction, wider pseudothorax and basal skirt in comparison with its pseudothorax, typical wing, and undulated apical cone.

***Longtanella turrita*** Xiao & Suzuki sp. nov.

(Figs 3F, S3P–U)

2016 *Longtanella zhengpanshanensis* Sheng & Wang; Shi *et al.*: fig. 4.13.

2020 *Longtanella* sp. A; Ito: fig. 6A.

Diagnosis. *Longtanella* with turreted shell.

Derivation of name. Latin adjective, *turritus*, -a, -um, meaning having a tower.

Material. Holotype, Fig. S3R, specimen ST1-3_052; paratype, Fig. S3Q, specimen ST1-3_048. Six specimens were examined by SEM and six of them are illustrated.

Measurements (μm). Height of shell 131–294 (mean 216); height of AC 17–33 (mean 21); width of the basal AC 17–26 (mean 22); height of PPA 33–66 (mean 44); height of PX 26–36 (mean 32); width of PX 35–50 (mean 41); height of PA 141–209 (mean 166); based on six specimens.

Occurrences. Middle Kungurian–Roadian; Gufeng and Bancheng Formation of South China.

Description. Shell tower-like, bilaterally symmetrical or slightly asymmetric, consisting of tiny apical cone, small pseudothorax, constricted pre-pseudoabdomen, and pseudoabdomen with culmina and crenellae. PX spherical, moderately inflated. PPA cylindrical and slim, 1.3–1.8 times longer than the height of PX. PA terraced, composed of 2–4 culmina which increase downwards, the first one shortest and sharply strictured. Flaps vertically or little bit obliquely extending from apertural margin downward.

Remarks. This new species was once identified as *Longtanella zhengpanshanensis* Sheng & Wang, 1985 by Shi *et al.* (2016). Reinvestigating the topotypes, Ito (2020) confirmed no intermediate forms between *L. zhengpanshanensis* in the sense of Shi *et al.* (2016) and *L. turrita* sp. nov..

***Longtanella kushidango*** Xiao & Suzuki sp. nov.

(Figs 3A, S4A–N)

1987 *Pseudoalbaillella lomentaria* Ishiga & Imoto; Niko *et al.*: pl. 1, figs 3–6.

1992 *Longtanella mengshengensis* Feng [nomen nudum]; Feng: pl. 1, figs 9, 10.

1992 *Albaillella* sp.; Hada *et al.*: fig. 4F.

1992 *Parafollicucullus lomentaria* Ishiga & Imoto; Harms & Murchey: pl. 1, fig. B.

1993 *Pseudoalbaillella lomentaria* Ishiga & Imoto; Li & Bian: 416, pl. 1, fig. 12.

1993 *Pseudoalbaillella lomentaria* Ishiga & Imoto; Sashida *et al.*: fig. 6.5.

1994 *Pseudoalbaillella lomentaria* Ishiga & Imoto; Wang *et al.*: 181, pl. 1, figs 12, 13.

1995 *Pseudoalbaillella lomentaria* Ishiga & Imoto; Sashida: 39, fig. 5.18.

1997 *Pseudoalbaillella lomentaria* Ishiga & Imoto; Basir Jasin & Che Aziz: 331, pl. 1, fig. 1.

1997 *Pseudoalbaillella* *annulata* Ishiga; Rudenko & Panasenko: pl.1, fig. 1

1997 *Pseudoalbaillella* cf. *lomentaria*; Rudenko & Panasenko: pl.1, fig. 2.

1998 *Pseudoalbaillella lomentaria* Ishiga & Imoto; Sashida *et al.*: fig.11. 16, 17.

2004 *Pseudoalbaillella* aff. *lomentaria*; Hori: pl. 1, figs. 25, 32.

2006 *Pseudoalbaillella lomentaria* Ishiga & Imoto; Shimakawa & Yao: pl. 1, figs 12, 13.

2012 *Pseudoalbaillella nodosa* Ishiga; Wang *et al.*: 47, pl. 11, figs 5, 6, 8.

2018 *Pseudoalbaillella* sp. cf. *P. lomentaria*; Udchachon *et al.*: 1460, figs 7h, 7k.

Diagnosis. *Longtanella* with cylindrical tube-like pseudothorax and pseudoabdomen with three to four culmina.

Derivation of name. Latinized word from Japanese, meaning a bunch of vertically arrayed three pasted rice balls of Japanese traditional sweet.

Material. Holotype, Fig. S4E, specimen ST9-1_i005; paratype, Fig. S4H, specimen ST9-1_i006. 17 specimens were examined by SEM and 14 of them are illustrated.

Measurements (μm). Height of shell 233–344 (mean 295); height of AC 56–98 (mean 81); width of the basal AC 40–61 (mean 51); height of PPA 17–39 (mean 29); height of PX 44–69 (mean 54); width of PX 78–107 (mean 93); height of PA 104–167 (mean 136); based on 16 specimens.

Occurrences. Middle Kungurian–Wordian; Gufeng and Bancheng Formation of South China; the Mizuyagadani Formation of the Hida-gaien Belt, Chichibu composite belts, Japan; Malaysia; Thailand; Sylvester Allochthon in the Canadian Cordillera, Canada; British Columbia, the Samarka Terrane in the Primorye, Far East Russia.

Description. Shell inflated and kushidango-like outline in shape. It consists of weakly undulated apical cone, inflated pseudothorax, short pre-pseudoabdomen, and pseudoabdomen with well-developed culmina and crenellae. AC usually with 3–5 segments but sometimes without segments (e.g. Fig. S4N). The total height of AC about 1/4 to 1/3 height of the shell. PX spherical, strongly inflated without wings, about 2/5 height of the PA. PPA short, about 1/2 height of the PX. PA composed of two inflated culmina,culmina of the same width of the pseudothorax; these culmina and crenellae oblique to the pseudothorax in some specimens, probably corresponding to the anatomical dorsal or ventral side (e.g. Fig. S4C–E). Posterior waist between the lower segment and basal skirt is narrower than segmental waists. BS similar to the segmental waist in width, but much narrower than the culmen.

Remarks. This new species is commonly illustrated worldwide (see occurrence) but has been confused with *Parafollicucullus lomentarius* (Ishiga & Imoto, 1980). However, *Pa. lomentarius* is fundamentally different from *L. kushidango* sp. nov. by having wings. *Longtanella mengshengensis* Feng, 1992 (pl. 1, figs 9, 10 in Feng 1992) is a *nomen nudum*, as we mentioned earlier. Rudenko & Panasenko (1997) illustrated *L. kushidango* sp. nov. as ‘*Pseudoalbaillella annulata*’ on pl. 1, fig. 1, probably because of the many culmina in the pseudoabdomen in their specimen.

***Longtanella laxiflexus*** Xiao & Suzuki sp. nov.

(Figs 3G, S4Q–U)

2004 *Pseudoalbaillella elegans* Ishiga & Imoto; Hori: pl. 4, figs 43, 44.

2004 *Pseudoalbaillella chilensis* Ling & Forsythe; Yao *et al.*: pl. 1, fig. 5.

2006 *Pseudoalbaillella chilensis* Ling & Forsythe; Shimakawa & Yao: pl. 1, fig. 5.

2012 *Pseudoalbaillella elegans* Ishiga & Imoto; Wang *et al.*: 44, pl. 11, fig. 10.

Diagnosis. *Longtanella* with weakly winding pseudoabdomen.

Derivation of name. The species name ‘*laxiflexus*’, derived from the Latin adjective ‘*laxus*’(-a, -um) + masculine noun *flexus* (-us, m), meaning ‘weak winding’.

**Material.** Holotype, Fig. S4U, specimen ST1-2_i011; paratype, Fig. S4Q, specimen 13ST9-1_083. Five specimens were examined by SEM and all of them are illustrated.

Measurements (μm). Height of shell 265–434 (mean 321); height of AC 50–102 (mean 75); width of the basal AC 33–60 (mean 47); height of PPA 27–69 (mean 40); height of PX 34–75 (mean 48); width of PX 60–82 (mean 69); height of PA 119–192 (mean 158); based on five specimens.

Occurrences. Middle Kungurian–Roadian; Bancheng Formation of South China and the Chichibu composite belts of Japan.

Description. Shell smooth, consisting of apical cone, rhombic pseudothorax, pre-pseudoabdomen and winding pseudoabdomen. AC conical and straight, about 1.5 times longer than PX. PX rhombic with no wings or wing-like rudiments. PPA has no obvious constriction, so PA is unable to be separated from PPA in the most cases with a few exceptions (Fig. S4U). PPA forms an annular ridge. PA long, constant in width without undulation, distinctly winding and forming an ‘S’ shape; the concave of posterior waist is on the ventral side. BS extends obliquely downwards.

Remarks. This new species has been confused with *Curvalbaillella chilensis* and *Curvalbaillella elegans* (Hori 2004; Yao *et al.* 2004; Shimakawa & Yao 2006; Wang *et al.* 2012). The new species is similar to *C. elegans* in the long and smooth pseudoabdomen without undulation, but is distinguished from the former by the longer pseudothorax and winding pseudoabdomen. The winding pseudoabdomen in the new species also resembles that in *C. chilensis*. Referred to morphological variations in *Curvalbaillella* illustrated in Kuwahara (1992, pl. 1), any *Curvalbaillella* specimens curve once only and possess distinctive wings on the pseudothorax. It differs from *Longtanella*? sp. 3 (Fig. S4O, P) in the smooth pseudoabdomen.

***Longtanella* *follicucullinoides*** Xiao & Suzuki sp. nov.

(Fig. S5A–G)

1989 *Pseudoalbaillella* *simplex* Ishiga & Imoto; Isozaki & Tamura, figs. 4.11, 4.12.

2005 *Pseudoalbaillella* sp. cf. *P. simplex* Ishiga & Imoto; Kametaka *et al.*: figs 4.8–4.14.

Diagnosis. *Longtanella* with long conical apical cone which smoothly continues from the apex to the slightly inflated pseudothorax and straight pseudoabdomen.

Derivation of name. Similar to the genus *Follicucullus*.

Material. Holotype, Fig. S5F, specimen ST1-2_i007; paratype, Fig. S5G, ST1-2_i022. Seven specimens were examined by SEM and all of them are illustrated.

Measurements (μm). Height of shell 185–329 (mean 216); height of AC 81–102 (mean 91); width of the basal AC 45–60 (mean 49); height of PPA 23–38 (mean 32); height of PX 43–55 (mean 50); width of PX 67–82 (mean 74); height of PA 18–149 (mean 51); based on seven specimens.

Occurrences. Middle Kungurian–Roadian; Bancheng Formation of South China; Nagato Tectonic zone and North Kitakami Terrane in Japan.

Description. Shell gracile and straight, consisting of a long apical cone, slightly inflated pseudothorax and cylindrical pseudoabdomen. Pre-pseudoabdomen generally absent. AC sturdy, conical without undulation, sometimes slightly curving to the ventral side. PX inflated with no wings, usually 1/2 of the height of AC. PPA, if present, cylindrical, similar to the width of PX. PA long, constant in width without undulation, about 1/2 of the height of shell. There develops a constriction between PPA and PA (see Fig. S5F), and the shell usually divided into two parts along this constriction.

Remarks. The more complete specimen in Fig. S5F shows that this morphospecies is gracile and weak in the boundary of the PPA and PA. This species may sometimes be confused with poorly-preserved specimens of *Curvalbaillella elongatus* (Ishiga & Imoto, 1980), *Parafollicucullus eurasiaticus* (Kozur & Mostler, 1989), *Parafollicucullus lanceolatus* (Ishiga and Imoto in Ishiga *et al.*, 1982), *Parafollicucullus lomentarius*, *Parafollicucullus longicornis* (Ishiga & Imoto, 1980) and *Parafollicucullus simplex* (Ishiga & Imoto, 1980). The new species is easily distinguishable from all these species in lacking wings even if specimens are poorly preserved. In the context of no wings in the new species, poorly-preserved *Follicucullus* is also similar to this species, but the new species is also easily distinguishable from the latter by its asymmetric shape which makes it hard to distinguish dorsal or ventral side.

**Reference list**:

Afanasieva, M. S. 2000. *Atlas of Paleozoic Radiolaria of the Russian Platform*. Scientifc World, Moscow, 477 pp. [In Russian.]

Afanasieva, M. S. 2002. A new classification of Paleozoic Radiolaria. *Paleontologicheskii Zhurnal*, 2, 14–29. [In Russian with English abstract.]

Afanasieva, M. S. & Amon, E. O. 2006. *Radiolaria*. Russian Academy of Sciences, Paleontological Institute, Institute of Geology and Geochemistry, PIN RAS Moscow, 320 pp. [In Russian.]

Afanasieva, M. S., Amon, E. O., Agarkov, Yu. V. & Boltovskoy, D. S. 2005. Radiolarians in the Geological Record. *Paleontological Journal*, 39, 135–392.

Basir Jasin & Che Aziz, A. 1997. Lower Permian Radiolaria from the Pos Blau area, Ulu Kelantan, Malaysia. *Journal of Asian Earth Sciences*, 15, 327–339.

Belyansky, G. S., Nikita, A. P. & Rudenko, V. S. 1984. About Sebuchaz suite of Primorye. Pp. 43–57 in Z. N. Poyarkova (ed.) *New Data an Detail Biostratigraphy of Phanerozoic of Far East*. DVNC Akademii Nauk SSSR, Vladivostok. [In Russian.]

Caridroit, M., Danelian, T., O'Dogherty, L., Cuvelier, J., Aitchison, J. C., Pouille, L., Noble, P., Dumitrica, P., Suzuki, N., Kuwahara, K., Maletz, J. & Feng, Q. L. 2017. An illustrated catalogue and revised classification of Paleozoic radiolarian genera. *Geodiversitas*, 39, 363–417.

Catalano, R., Di Stefano, P. & Kozur, H. 1989. Lower Permian Albaillellacea (Radiolaria) from Sicily and their stratigraphic and paleogeographic significance. *Rendiconto dell’Accademia delle Scienze fsiche e matematiche, Serie IV*, 56, 1–24.

Cheng, Y. N. 1986. *Taxonomic Studies on Upper Paleozoic Radiolaria*. National Museum of natural Science, Taiwan, special Publication 1, 310pp.

Cordey, F. 1998. *Radiolaires des complexes d'accrétion de la Cordillère Canadienne (Colombie-Britannique)*. Geological Survey of Canada Bulletin 509, 209 pp. [In French with English summary.]

Deflandre, G. 1953. Radiolaires fossiles. Pp. 389–436 in P. P. Grassé (ed.) *Traité de Zoologie*. Masson, Paris.

De Wever, P., Dumitrica, P., Caulet, J. P., Nigrini, C. & Caridroit, M. 2001. *Radiolarians in the Sedimentary Record*. Gordon and Breach Science Publishers, Amsterdam, 533 pp.

Feng, Q. L. 1992. Permian and Triassic radiolarian biostratigraphy in south and southwest China. *Earth Science-Journal of China University of Geosciences*, 31, 51–62.

Hada, S., Salo, E., Takeshima, H. & Kawakami, A. 1992. Age of the covering strata in the Kurosegawa Terrane: dismembered continental fragment in southwest Japan. *Palaeogeography, Palaeoclimatology, Palaeoecology*, 96, 59–69.

Harms, T. A. & Murchey, B. L. 1992. Setting and occurrence of Late Paleozoic radiolarians in the Sylvester allochthon, part of a proto-Pacific ocean floor terrane in the Canadian Cordillera. *Palaeogeography, Palaeoclimatology, Palaeoecology*, 96, 127–139.

Holdsworth, B. K. 1969. Namurian Radiolaria of the genus *Ceratoikiscum* from Staﬀordshire and Derbyshire, England. *Micropaleontology*, 15, 221–229.

Holdsworth, B. K. & Jones, D. L. 1980. Preliminary radiolarian zonation for late Devonian through Permian time. *Geology*, 8, 281–285.

Hori, N. 2004. Permian radiolarians from chert of the Chichibu Belt in the Toyohashi district, Aichi Prefecture, Southwest Japan. *Bulletin of the Geological Survey of Japan*, 55, 287–301. [In Japanese with English abstract.]

Ishiga, H. 1982. Late Carboniferous and Early Permian radiolarians from the Tamba Belt, Southwest Japan. *Earth Science, Journal of the Association for the Geological Collaboration in Japan*, 36, 333–339.

Ishiga, H. 1983. 756. Morphological change in the Permian radiolaria, *Pseudoalbaillella scalprata* in Japan. *Transactions and proceedings of the Paleontological Society of Japan. New series*, 129, 1–8.

Ishiga, H. 1991. Description of a new *Follicucullus* species from southwest Japan. *Memoirs of the Faculty of Science, Shimane University*, 25, 107–118.

Ishiga, H. & Imoto, N. 1980. Some Permian radiolarians in the Tamba District, Southwest Japan. *Earth Science, Journal of the Association for the Geological Collaboration in Japan*, 34, 333–345.

Ishiga, H., Kito, T. & Imoto, N. 1982. Middle Permian radiolarian assemblages in the Tamba District and an adjacent area, southwest Japan. *Earth Science (Chikyu Kagaku)*, 36, 272–281.

Ishiga, H., Imoto, N., Yoshida, M. & Tanabe, T. 1984. Early Permian radiolarians from the Tamba Belt, Southwest Japan. *Earth Science (Chikyu Kagaku)*, 38, 44–52.

Isozaki, Y. & Tamura, H. 1989. Late Carboniferous and Early Permian radiolarians from the Nagato Tectonic Zone and their implication to geologic structure of the Inner Zone, Southwest Japan. *The Memoirs of Geological Society of Japan*, 33, 167–176. [In Japanese with English abstract.]

Ito, T. 2020. Taxonomic re-evaluation of the Permian radiolarian genus *Longtanella* Sheng and Wang (Follicucullidae, Albaillellaria). *Revue de Micropaléontologie*, 66, 100406.

Ito, T., Feng, Q. L. & Matsuoka, A. 2013. Radiolarian faunal change in the Middle Permian Gufeng Formation in the Liuhuang section, Chaohu, South China. *Science Reports of Niigata University (Geology)*, 28, 39–49.

Ito, T., Feng, Q. L. & Matsuoka, A. 2015. Taxonomic significance of short forms of middle Permian *Pseudoalbaillella* Holdsworth and Jones, 1980 (Follicucullidae, Radiolaria). *Revue de Micropaléontologie*, 58, 3–12.

Ito, T., Kitagawa, Y. & Matsuoka, A. 2016. Middle and Late Permian radiolarians from chert blocks within conglomerates of the Kamiaso Unit of the Mino Terrane in Gifu Prefecture, central Japan. *Journal of the Geological Society of Japan*, 122, 249–259. [In Japanese with English abstract.]

Ito, T., Takahashi, K. U., Matsuoka, A. & Feng, Q. L. 2019. The Guadalupian (Permian) Gufeng Formation on the north margin of the South China block: a review of the lithostratigraphy, radiolarian biostratigraphy, and geochemical characteristics. *Paleontological Research*, 23, 261–280.

Kametaka, M., Nakae, S. & Kamada, K. 2005. Early Permian radiolarians from siliceous mudstone in the Rikuchu-Seki District, North Kitakami Terrane. *Bulletin of the Geological Survey of Japan*, 56, 237–243. [In Japanese with English abstract.]

Kametaka, M., Nagai, H., Zhu, S. Z. & Takebe, M. 2009. Middle Permian radiolarians from Anmenkou, Chaohu, Northeastern Yangtze platform, China. *Island Arc*, 18, 108–125.

Kashiwagi, K. & Isaji, S. 2015. Paleozoic and Mesozoic radiolarians from chert pebbles and cobbles of the Lower Cretaceous Choshi Group, Japan. *Natural History Research (Natural History Museum and Institute, Chiba)*, 13, 35–46.

Kozur, H. 1981. Albaillellidea (Radiolaria) aus dem Unterperm des Vorurals. *Geologisch-Paläontologische Mitteilungen Innsbruck*, 10, 263–274.

Kozur, H. & Mostler, H. 1989. Radiolarien und Schwammkleren aus dem Unterperm des Vorurals. *Geologisch-Paläontologische Mitteilungen Innsbruck*, 2, 147–275.

Kuwahara, K. 1992. Late Carboniferous to Early Permian radiolarian assemblage from Miyagawa area, Mie Prefecture, Japan. *News of Osaka Micropaleontologists,* *Special Volume*, 8, 1–7 [In Japanese with English abstract.]

Li, H. S. & Bian, Q. T. 1993. Upper Paleozoic Radiolaria of the Xijin Ulan -Gangqiqu Ophiolite Complex, Kekexili. *Geoscience-Journal of Graduate School, China University of Geosciences*, 7, 410–420. [In Chinese with English abstract.]

Ling, H. Y. & Forsythe, R. D. 1987. Late Paleozoic pseudoalbaillellid radiolarians from southernmost Chile and their geological significance. Pp. 253–260 in G. D. McKenzie (ed.) *Gondwana Six: Structure, Tectonics, and Geophysics*. *Geophysical Monograph Series 40*. John Wiley & Sons, Inc., New York.

Metcalfe, I., Spiller, F. C. P., Liu, B. P., Wu, H. R. & Sashida, K. 1999. The Palaeo-Tethys in Mainland East and Southeast Asia: contributions from radiolarian studies. Pp. 259–281 in I. Metcalfe (ed.) *Gondwana Dispersion and Asian Accretion*. IGCP321 Final Results Volume. A.A. Balkema, Rotterdam.

Muhammad Ashahadi, D., Basir Jasin & Mohd Shafeea L. 2016. Taksonomi Radiolaria dari genus *Pseudoalbaillella* berusia Perm dari Pos Blau, barat daya Kelantan, Semenanjung Malaysia. *Bulletin of the Geological Society of Malaysia*, 6, 13–21.

Nagai, H., Zhu, S. Z., Kametaka, M. & Wu C. Y. 1998. Preliminary report on Middle Permian radiolarians from the Gufeng Formation at Anmenkou, Chaohu City, Anhui Province, China. *Bulletin of the Nagoya University Furukawa Museum*, 14, 115–123.

Nakagawa, T. & Wakita, K. 2020. Morphological insights from extremely well-preserved *Parafollicucullus* (Radiolaria, Order Albaillellaria) from a probable Roadian (Guadalupian, middle Permian) manganese nodule in the Nishiki Group of the Akiyoshi Belt, Southwest Japan. *Paleontological Research*, 24, 161–177.

Nazarov, B. B. 1988. *Paleozoic radiolaria, Practical manual of microfauna of the USSR*. Radiolyarii Paleozoy 2, Nedra, Leningrad, 232 pp. [In Russian.]

Nestell, G. P. & Nestell, M. K. 2010. Late Capitanian (latest Guadalupian, Middle Permian) radiolarians from the Apache Mountains, West Texas. *Micropaleontology*, 56, 7–68.

Nestell, G. P. & Nestell, M. K. 2020. Roadian (earliest Guadalupian, Middle Permian) radiolarians from the Guadalupe Mountains, West Texas, USA. Part I: Albaillellaria and Entactinaria. *Micropaleontology*, 66, 1–50.

Nestell, G. P., Pope, J. P. & Nestell, M. K. 2012. Middle Pennsylvanian (Desmoinesian) Radiolaria from the Midcontinent of North America. *Micropaleontology*, 58, 217–257.

Nestell, K. M., Nestell, G. P. & Wardlaw, B. R. 2019. Integrated fusulinid, conodont, and radiolarian biostratigraphy of the Guadalupian (middle Permian) in the Permian Basin regions. Pp. 251–291 in S. C. Ruppel (ed.) *Anatomy of a Paleozoic Basin: the Permian Basin, USA, Vol. 1*. AAPG Memoir, 118.

Niko, S., Yamakita, S., Otoh, S., Yanai, S. & Hamada, T. 1987. Permian radiolarians from the Mizuyagadani Formation in Fukuji area, Hida Marginal Belt and their significance. *Journal of the Geological Society of Japan*, 93, 431–433. [In Japanese.]

Nishimura, K. & Ishiga, H. 1987. Radiolarian biostratigraphy of the Maizuru Group in Yanahara area, Southwest Japan. *Memoirs of the Faculty of Science, Shimane University*, 21, 169–188.

Noble, P. J., Aitchison, J. C., Danelian, T., Dumitrica, P., Maletz, J., Suzuki, N., Cuvelier, J., Caridroit, M. & O'Dogherty, L. 2017. Taxonomy of Paleozoic radiolarian genera. *Geodiversitas*, 39, 419–502.

Ormiston, A. R. & Babcock, L. 1979. *Follicucullus*, new radiolarian genus from the Guadalupian (Permian) Lamar Limestone of the Delaware Basin. *Journal of Paleontology*, 53, 323–334.

Petrushevskaya, M. G. 1984. On the classification of polycystine radiolarians. Pp. 64–87 in O. A. Scarlato, G. I. Poljansky, A. I. Zhamoida, A. N. Golikov, L. I. Kasinsova, S. B. Kruglikova & T. G. Lukina (eds) *Morphology, Ecology, and Evolution of Radiolarians*. Nauka, Leningrad. [In Russian.]

Rudenko, V. S. 1991. *Permskie Albaillellaria (Radiolyarii) primor'ya i ikh biostratigraficheskoe znachenie. Dal'nevostochoe Oredena Trudovogo Krasnogo Znameni Otdelenie*. Dal'nevostochnyiy Geologicheskiy Institut, Akademiya Nauk SSSR, Vladiovostok, 27 pp. [In Russian.]

Rudenko, V. S. & Panasenko, E. S. 1990. Permian Albaillellaria (Radiolaria) of the Pantovyi Creek sequence in Primorye. Pp. 181–193 in G. I. Tisarena (ed.) *New Data on Paleozoic and Mesozoic Biostratigraphy of the South Far East*. USSR Academy of Sciences, Far-Eastern Branch, Vladivostock. [In Russian.]

Rudenko, V. S. & Panasenko, E. S. 1997. Biostratigraphy of Permian deposits of Sikhote-Alin based on radiolarians. Pp. 73–84 in A. Baud, I. Popova, J. M. Dickins, S. Lucas, Y. Zakharov (eds) *Late Paleozoic and Early Mesozoic Circum-Paciric Events: Biostratigraphy, Tectonic and Ore Deposits of Primoryie (Far East Russia)*. IGCP Project 272. Mémoires de Géologie (Lausanne), 30.

Saesaengseerung, D., Agematsu, S., Sashida, K. & Sardsud, A. 2009. Discovery of Lower Permian radiolarian and conodont faunas from the bedded chert of the Chanthaburi area along the Sra Kaeo suture zone, eastern Thailand. *Paleontological Research*, 13, 119–138.

Sashida, K. 1995. Late Carboniferous and Early Permian radiolarian biostratigraphy in the chert block embedded in the Jurassic Kawai Formation, Kanto Mountains, central Japan. *Annual Report of the Institute of Geoscience, the University of Tsukuba*, 21, 33–40.

Sashida, K., Igo, H., Hisada, K. I., Nakornsri, N. & Ampornmaha, A. 1993. Occurrence of Paleozoic and Early Mesozoic Radiolaria in Thailand (preliminary report). *Journal of Southeast Asian Earth Sciences*, 8, 97–108.

Sashida, K., Igo, H., Adachi, S., Ueno, K., Nakornsri, N. & Sardsud, A. 1998. Late Paleozoic radiolarian faunas from northern and northeastern Thailand. *Science Reports of the Institute of Geosciences, University of Tsukuba, section B*, 19, 1–27.

Sheng, J. Z. & Wang, Y. J. 1985. Fossil Radiolaria from Kufeng Formation at Longtan, Nanjing. *Acta Palaeontologica Sinica*, 24, 171–180.

Shi, L., Feng, Q. L., Shen, J., Ito, T. & Chen, Z. Q. 2016. Proliferation of shallow-water radiolarians coinciding with enhanced oceanic productivity in reducing conditions during the Middle Permian, South China: evidence from the Gufeng Formation of western Hubei Province. *Palaeogeography, Palaeoclimatology, Palaeoecology*, 444, 1–14.

Shimakawa, M. & Yao, A. 2006. Lower–Middle Permian radiolarian biostratigraphy in the Qinzhou area, South China. *Journal of Geosciences, Osaka City University*, 49, 31–47.

Spiller, F. C. P. 2002. Radiolarian biostratigraphy of Peninsular Malaysia and implications for regional palaeotectonics and palaeogeography. *Palaeontographica Abteilung A Palaeozoology – Stratigraphy*, 266, 1–91.

Udchachon, M., Thassanapak, H. & Burrett, C. 2018. Early Permian radiolarians from the extension of the Sa Kaeo Suture in Cambodia–tectonic implications. *Geological Magazine*, 155, 1449–1464.

Ujiié, H. & Oba, T. 1991. Geology and Permo-Jurassic Radiolaria of the Iheya Zone, Innermost Belt of the Okinawa Islands region, middle Ryukyu island arc, Japan. Part 1: Geology and Permian Radiolaria. *Bulletin of College Science, University of Ryukyus*, 51, 35–55.

Wang, R. J. 1993. Fossil Radiolaria from Kufeng Formation of Chaohu, Anhui. *Acta Palaeontologica Sinica*, 32, 442–457. [In Chinese with English abstract.]

Wang, Y. J. & Qi, D. L. 1995. Radiolarian fauna of the Kuhfeng Formation in southern part of Jiangsu and Anhui provinces. *Acta Micropalaeontologica Sinica*, 12, 374–387.

Wang, Y. J., Cheng, Y. N. & Yang, Q. 1994. Biostratigraphy and systematics of Permian radiolarians in China. *Palaeoworld*, 4, 172–202.

Wang, Y. J., Luo, H. & Yang, Q. 2012. *Late Paleozoic radiolarians in the Qinfang area, southeast Guangxi.* University of Science and Technology of China, Anhui, 127 pp. [In Chinese with English abstract.]

Wu, H. R., Xian, X. Y. & Kuang, G. D. 1994. Late Paleozoic radiolarian assemblages of southern Guangxi and its geological significance. *Scientia Geologica Sinica*, 29, 339–345. [In Chinese with English abstract.]

Xia, W. C. & Zhang, N. 1998. Early to Middle Permian radiolarians from the Kuhfeng Formation in southeastern Guangxi, South China. *Earth Science, Journal of the Association for the Geological Collaboration in Japan*, 52, 188–202.

Xiao, Y. F., Suzuki, N., He, W. H., Benton, M. J., Yang, T. L. & Cai, C. Y. 2020. Verifiability of genus-level classification under quantification and parsimony theories: a case study of follicucullid radiolarians. *Paleobiology*, 46, 337–355.

Yao, A., Kuwahara, K., Ezaki, Y., Liu, J. B. & Hao, W. C. 2004. Permian radiolarians from the Qinfang Terrane, South China, and its geological significance. *Journal of Geosciences, Osaka City University*, 47, 71–83.

**Supplement 6. The character data set used for phylogenetic tree of Follicucullidae.**

**Supplement 6-1.** List of species and the codes of each character for each species. The codes and states of the 49 characters are explained in Supplement 6-2.


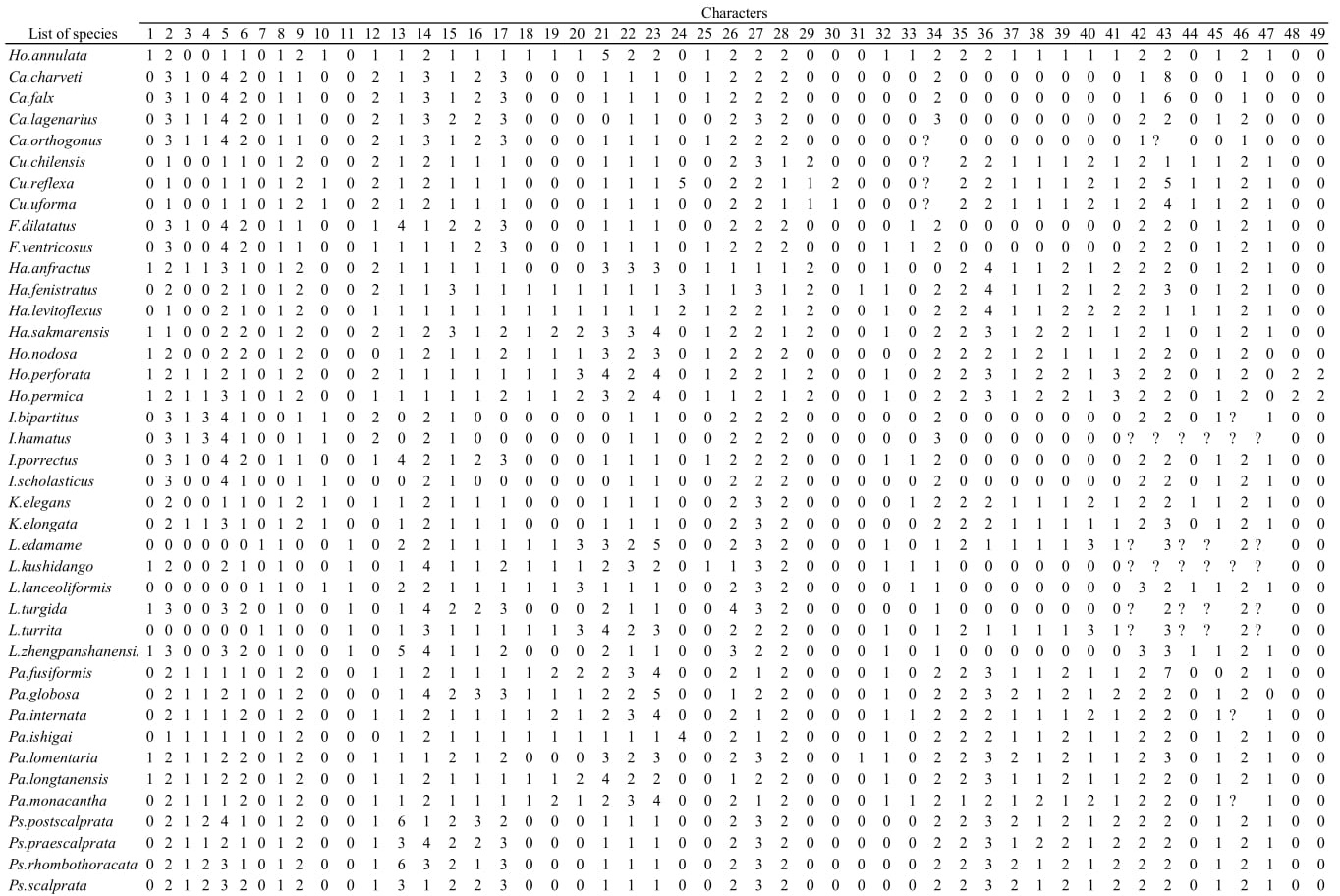


Abbreviations: *Ho.*, *Holdsworthella*; *Ha.*, *Haplodiacanthus*; *Ps.*, *Pseudoalbaillella*; *Pa.*, *Parafollicucullus*; *L.*, *Longtanella*; *K.*, *Kitoconus*; *Ca.*, *Cariver*; *Cu.*, *Curvalbaillella*; *F.*, *Follicucullus*; *I.*, *Ishigaconus*.

**Supplement 6-2.** The codes and states of the 49 characters.


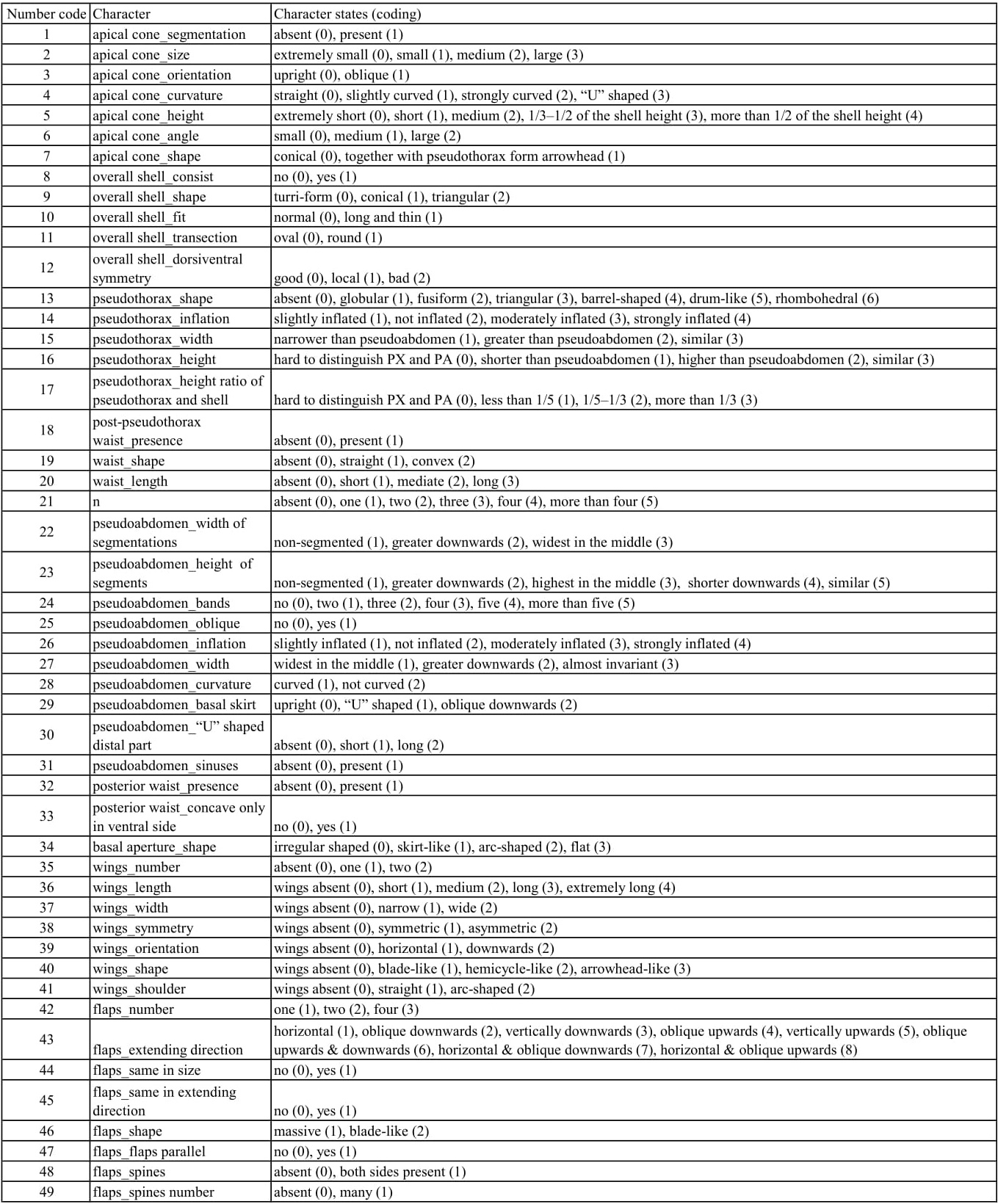


**Supplement 7. Meta dataset for CA. 1: occurrence, 0: absent.**


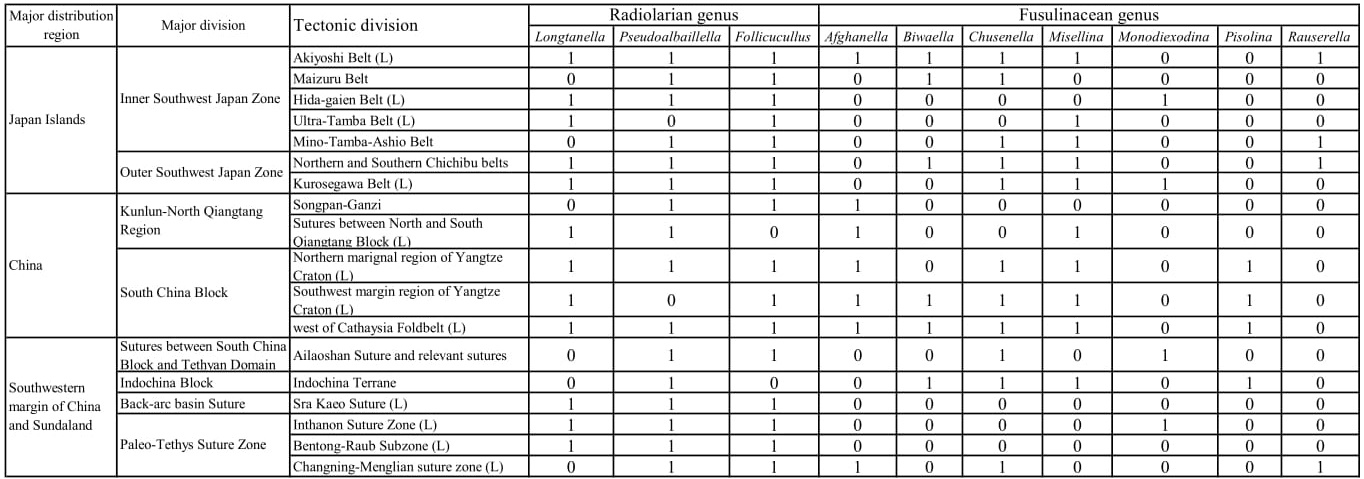

Supplement: Supplementary file 1 — Supplementary Information [file 41598_2021_86262_MOESM1_ESM.docx]
